# Gut Microbiota of *Ostrinia nubilalis* Larvae Degrade Maize Cellulose

**DOI:** 10.3389/fmicb.2022.816954

**Published:** 2022-04-11

**Authors:** Junfeng Li, Siran Wang, Jie Zhao, Zhihao Dong, Tao Shao

**Affiliations:** Institute of Ensiling and Processing of Grass, College of Agro-grassland Science, Nanjing Agricultural University, Nanjing, China

**Keywords:** European corn borer, gut microbiota, cellulose, degradation, microbial consortia

## Abstract

Most arthropod guts harbor diverse microbiota for symbiotic digestion. The European corn borer (ECB), *Ostrinia nubilalis* (Hübner), is a devastating pest that feeds the lignocellulose-rich tissues of maize plants. However, the potential role of ECB gut microbes in degrading maize cellulose remains largely unexplored. Here, we investigated the gut microbiota of ECB fed with different diets and their potential function in maize lignocellulose degradation. The diversity and composition of gut bacterial communities varied dramatically between the ECB larva fed with artificial diets (ECB-D) and maize plants (ECB-M). Draft genomes of the microbial consortia from ECB-D and ECB-M showed that the principal degraders of cellulose mainly belonged to Firmicutes or Proteobacteria and they were primarily found in the midgut. The cellulolytic microbial consortia contained genes encoding various carbohydrate-active enzymes (CAZyme). Furthermore, scanning electron microscopy revealed significant breakdown of lignocellulose in maize treated by the two microbial consortia for 9 days *in vitro*. Metabolomic analyses show that maize particles treated by two microbial consortia generate distinctive metabolomic profiles, with enrichment for different monosaccharides (i.e., Glucose, Rhamnofuranose, Isomaltose, and Cellobiose) and amino acids (i.e., Threonine, Histidine, and Lysine). The results indicated that the diet of the host impacted the composition and function of its gut microbiota and ECB exploited specific gut microbes to digest maize lignocellulose with distinctive products. Our study provides valuable microbiota resources for lignocellulose bioconversion.

## Introduction

Cellulose is a renewable and abundant biomass resource widely distributed in all higher plants (Varner and Lin, 1989; Naik et al., 2010). The long chains of cellulose polymers are linked together to shape into microfibrils arranged in uniform forming a crystalline structure that cause cellulose less susceptible to biodegradation (Ali et al., 2020). Cellulose degradation is the most critical step for its utilization, but current methods (e.g., physical and chemical) employed for processing cellulose are high in cost and cause environmental pollution (Kumar et al., 2009; Stephen et al., 2012). Many phytophagous insects, such as termites, beetles, and wasps, possess efficient microscale bioconversion systems of cellulose in their bodies (Sun and Scharf, 2010; Luo et al., 2019; Zheng et al., 2019). Gut microbiota that produces cellulases to digest lignocellulose into sugars provides a major energy source to these species (Kundu et al., 2019). These systems can contribute valuable information about utilizing plant cellulosic biomass as a sustainable energy source (Watanabe and Tokuda, 2010).

While the vast majority of Lepidoptera are also herbivores, their gut microbes show relatively low diversity and can be impacted by environmental factors compared to other phytophagous insects (Priya et al., 2012; Yun et al., 2014; Vilanova et al., 2016). It has been previously demonstrated that many caterpillars carry diverse gut bacteria with essential functions for the host (Prem Anand et al., 2010; Belda et al., 2011), but considering their simple gut morphology and rapid digestive throughput, it remains disputed whether microbes persist in the host gut and contribute to host phytophagy (Jones et al., 2019). For example, a recent study showed that wild leaf-feeding caterpillars lack a resident symbiotic gut microbiome (Hammer et al., 2017). Furthermore, although many caterpillars consume cellulose-rich plants (e.g., maize and rice), the composition and role of gut microbiota in their feeding and digestion remain limited. The European corn borer (ECB), *Ostrinia nubilalis* (Hübner) (Lepidoptera: Crambidae), is a devastating pest that feeds on the leaves and stalks of maize, which contain polysaccharides including cellulose, hemicellulose, and lignin (Dhugga, 2007; Krumm et al., 2008). Like other insects thriving on a cellulose-rich diet, ECB host a diverse bacterial community in their gut (Belda et al., 2011), of which some produce cellulases (Vilanova et al., 2012). However, the role of gut bacteria in their digestion of cellulose has received little attention. Although no convincing evidence of maize cellulose digestion through the gut microbiota of ECB has been reported, developments in next-generation sequencing technologies provide opportunities to better characterize the microbiome and its associated genomic resources in non-model organisms.

In this study, our objectives were to (i) examine the gut microbiota of ECB fed with different diets and (ii) investigate the potential function of their gut bacteria in maize lignocellulose degradation. To achieve these aims, we firstly isolate and identify cellulose-degrading microbial consortia from the gut of ECB larvae that were fed artificial diets and maize plants, respectively. We also analyze the draft genomes of the cellulolytic microbial consortia from the gut of ECB larvae and identify genes encoding the carbohydrate active enzymes (CAZymes). Finally, we estimate the lignocellulose degradation efficiencies of the cellulolytic microbial consortia *in vitro* and the metabolomic profiles of maize straw after being exposed to each microbial consortium.

## Materials and Methods

### Insect Collection and Maintenance

The ECB, *Ostrinia nubilalis* (Hübner), was originally collected from maize plants (*Zea mays* L.) in Nanjing, China (N32°02′, E118°52′) in 2019. About 100 third-instar larvae were split into two groups and fed separate diets: an artificial diet (designated as ECB-D), and maize plants (*Z. mays* strain Nannong3; designated as ECB-M). An artificial diet was prepared as previously described by Vilanova et al. (2016). Larvae were reared at 25 ± 1°C under 70% relative humidity and 15 h light: 9 h dark conditions. After four generations, third-instar larvae from both the ECB-D and ECB-M populations were collected for dissecting the gut and subsequently experiments.

### Analysis of the European Corn Borer Intestinal Microbiota

#### DNA Extraction, Library Construction, and Sequencing

We randomly selected six individuals from each ECB-D and ECB-M population. The guts of individual ECB were dissected in sterilized phosphate-buffered saline (PBS) and surface sterilized using 75% ethanol and sterile dH_2_O. Total DNA was extracted from the individual gut using the QIAGEN DNeasy Kit (Germany) according to the manufacturer’s protocol. Amplicons were obtained using the universal primers 341F (5′-CCTAYGGGRBGCASCAG-3′) and 806R (5′-GGACTACNNGGGTATCTAAT-3′), extracted from 2% agarose gels, and purified using the AxyPrep DNA Gel Extraction Kit (Axygen Biosciences, United States) and quantified using QuantiFluor™-ST (Promega, United States). Purified PCR products were quantified with Qubit^®^3.0 (Life Invitrogen). Amplicons from DNA samples and the negative control were sequenced with an Illumina HiSeq 2500 platform (Shanghai BIOZERON Co., Ltd., China).

#### Microbiota Analysis

A total of 521239 raw reads were obtained. Raw data were processed using QIIME version 1.7.0 (Caporaso et al., 2010). Briefly, raw *fasta* files were first demultiplexed using in-house Perl scripts to process barcode sequence information for each sample with the following criteria: (i) The 250 bp reads were truncated at any site receiving an average quality score < 20 over a 10 bp sliding window, discarding the truncated reads that were shorter than 50 bp. (ii) exact barcode matching, 2 nucleotide mismatches in primer matching and reads containing ambiguous characters were removed. (iii) only sequences that overlap for longer than 10 bp were assembled according to their overlapping sections. Reads which could not be assembled were discarded. The sequences were assigned to operational taxonomic units (OTUs) at the 97% similarity threshold using UPARSE version 7.1^1^ (Edgar, 2013) and chimeric sequences were identified and removed using UCHIME (Edgar et al., 2011). Prior to microbial analysis, taxa with < 0.1% abundance summed across all samples were removed. Microbial analysis was performed by MicrobiomeAnalyst^2^ (Dhariwal et al., 2017). Non-parametric *t*-tests were used to detect significant differences in the Shannon diversity index between the gut bacterial community of ECB larvae fed on an artificial diet and maize plants. Variation in bacterial taxonomic composition among samples was visualized using principal coordinates analyses (PCoA). Additionally, a linear discriminant analysis of effect size (LEfSe) method (Segata et al., 2011) was used to determine significant differences between bacterial communities of two treatments using with α = 0.05 for the initial Kruskal–Wallis test and applying a linear discriminant analysis (LDA) score threshold of 2.0. The metabolic potential of the microbiomes was predicted with PICRUSt (Langille et al., 2013).

### Isolation and Enrichment of Cellulolytic Microbial Consortia

#### Isolation and Identification of Cellulolytic Microbial Consortia

The 50 guts dissected from ECB larvae were blended, homogenized, and filtered using 10 μm syringe filters. Subsequently, 1 mL suspension was immediately plated on Luria-Bertani (LB) media with 5 g/L carboxymethyl cellulose (CMC) agar and incubated at 30°C and 150 rpm for cellulolytic bacteria screening. Congo red dye was used to screen for cellulose-degrading bacteria, as described by Teather and Wood (1959). Two cellulolytic microbial consortia were obtained, one from the gut microbiota of ECB larva fed with an artificial diet (designated as BI-D), and another from those fed with maize plants (designated as BI-M) (Supplementary Figure 1).

#### Genomic DNA Extraction and Genome Sequencing

Total genomic DNA was extracted from two cellulolytic microbial consortia BI-D and BI-M using the Bacteria DNA Kit (OMEGA), and quality control was subsequently carried out on the purified DNA samples. Genomic DNA was quantified using the TBS-380 fluorometer (Turner BioSystems Inc., Sunnyvale, CA, United States), and DNA libraries were constructed under standard procedures. The qualified Illumina pair-end library would be used for Illumina NovaSeq 6000 sequencing (Shanghai BIOZERON Co., Ltd., Shanghai, China). Raw reads were trimmed and quality controlled by Trimmomatic (version 0.36^3^). Filtered data were used for downstream analyses. We used *ab initio* prediction methods to obtain gene models for microbial consortia. Gene models were identified using GeneMark.

#### CAZyme Assignment of Genome Sequences

Protein sequences were analyzed using the dbCAN CAZyme annotation algorithm^4^ with default parameters to determine the carbohydrate active enzymes (CAZymes) in the two cellulolytic microbial consortia (BI-D and BI-M).

#### Fluorescence *in situ* Hybridization

To investigate the localization of the dominant genera in the two microbial consortia within the digestive tract of ECB larvae, fluorescence in situ hybridization (FISH) was performed with genus-specific probes (Supplementary Table 1). Gut tissue (i.e., foregut, midgut, and hindgut) for each sample were washed twice with PBS. Then, samples were fixed in 1 mL of 4% paraformaldehyde for 30 min, followed by two washes in 1 mL of PBS for 2 min. Hybridization was performed for 12 h at 42°C using 1 mL of hybridization buffer [20 mM Tris-HCl pH 7.4, 0.02% sodium dodecyl sulphate (SDS), 0.9 M NaCl, 5 mM ethylenediaminetetraacetic acid (EDTA), 60% formamide] and 0.002 mL of the probe. After hybridization, samples were washed twice at 46°C for 30 min in 1 mL of hybridization wash buffer. Finally, samples were viewed under a ZEISS LSM 700 confocal microscope (Carl Zeiss, Germany). Figures were processed with PHOTOSHOP 4.0 software (Adobe Systems Inc., San Jose, CA, United States). A FISH reaction without a probe was performed as a negative control.

### Maize Degradation by Microbial Consortia *in vitro*

#### Experimental Design

Experiments were carried out on six independent biological replicates of each treatment. Maize straw was powdered and milled through a 0.5 mm screen. The microbial consortia (5 mL BI-D or BI-M) were separately cultured in 100 mL of the liquid medium at a pH of 8.0, with a temperature of 37°C and 150 rpm for 9 days, respectively. The culture medium comprised of maize straw powder 5 g/L, yeast extract 0.5 g/L, malt extract 0.5 g/L, tryptone 0.5 g/L, NaCl 0.5 g/L, KH_2_PO_4_ 0.2 g/L, MgSO_4_⋅7H_2_O 0.13 g/L, and CaCl_2_ 0.5 g/L. The negative control had only a sterile culture medium. The reaction products were centrifuged at 13,000 rpm for 10 min. The hydrolysate-containing supernatant was stored at −80°C for the determination of cellulolytic activity and metabolomic analysis. Endoglucanase, β-glucosidase and exoglucanase activity were determined using the Solarbio assay kit (Solarbio, Beijing, China). The obtained deposit was dried and weighed to determine levels of cellulose, hemicellulose, and lignin content as described by Van Soest et al. (1991).

#### Scanning Electron Microscopy

Surface morphology of the untreated and 9-day treated maize straw powder samples were observed and analyzed using scanning electron microscopy (SEM) as described by Karnovsky (1965). Briefly, maize particles from each treatment were fixed overnight at 4°C in 2.5% glutaraldehyde (Electron Microscopy Sciences, United States) and 0.1 M sodium phosphate buffer (pH 7.2). Fixed samples were washed three times in 0.1 M sodium phosphate buffer (pH 7.2) and were stepwise dehydrated with 30, 50, 70, 80, 90, and 100% concentrations of ethanol, followed by a final treatment using 100% acetone. Then, specimens were critical point dried and coated with gold particles in the Technics Hummer VI Sputter Coat Unit (Anatech, United States). After gold-sputtering, the samples were observed under SEM (SU-8010, JEOL Ltd., Japan) at an acceleration voltage of 5.0 kV.

#### Metabolomic Analysis

Six biological replicates of each treatment were shipped on dry ice to Shanghai BIOZERON Co., Ltd. (Shanghai, China) for metabolomic analysis. The metabolites were extracted in 80% methanol by vortexing for 10 min, centrifuged, and then the supernatant was filtered using a 0.22 μm membrane (J&K Scientific, Beijing, China). One μL of each sample was loaded and analyzed using liquid chromatography-mass spectrometry (LC-MS). Non-targeted metabolite profiling was carried out on a Thermo Ultimate 3000 system equipped with an ACQUITY UPLC^®^ HSS T3 (150 mm × 2.1 mm, 1.8 μm, Waters). Statistical differences between samples were investigated with a one-way ANOVA, followed by Tukey’s HSD *post hoc* test and FDR correction using MetaboAnalyst (Xia and Wishart, 2016).

## Results

### Gut Microbiota of European Corn Borer Larvae Is Determined by Diet

**FIGURE 1:**
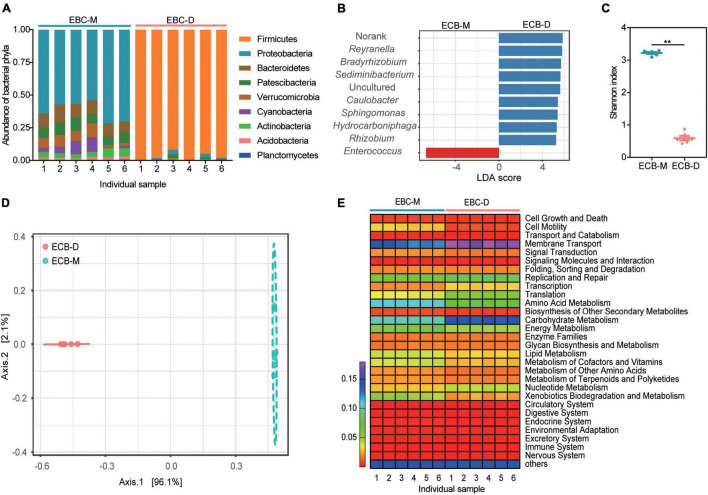
Gut microbial diversity and community composition in the ECB larvae fed with an artificial diet (ECB-D) or maize plants (ECB-M). **(A)** Relative abundance of microbiota in both strains at the phylum level. The number of *x*-axis indicates individual gut sample. **(B)** Linear discriminant analysis effect sizes (LEFSe) for the top 10 bacterial genus that differed significantly in relative abundance between ECB-D and ECB-M. **(C)** α-diversity comparison based on the Shannon diversity index, using a *t*-test to determine significant differences (**P < 0.01). Horizontal lines indicate the mean (± SE) of biological replicates. **(D)** Principal coordinate analysis (PCoA) plot generated using OTU metrics based on Bray-Curtis distance. The variation explained by the PCoA axes is given in parentheses. **(E)** Heatmap showing the main function of microbiota present in the larval gut of ECB-D and ECB-M with different abundance.

A total of 350225 sequences were obtained from the 12 samples sequenced for bacterial 16S rRNA gene amplicons, with an average of 29185 ± 887 (standard deviation) reads per sample after quality filtering and removal of chimeric sequences. All the sequences were classified into 61 OTUs (>0.1% of all sequences) at 97% sequence identity, which belonged to 9 phyla, 13 classes, 24 orders, 30 families, and 34 genera. Overall, the phylum Firmicutes was the most abundant in all samples (50%), followed by Proteobacteria (31%), Bacteroidetes (5%), Patescibacteria (5%). The gut bacterial community composition differed significantly between ECB-M and ECB-D. The genus *Enterococcus* (96.89%) within Firmicutes was dominant in ECB-D, whereas those from ECB-M were *Reyranella* (12.92%), *Bradyrhizobium* (10.29%), *Sediminibacterium* (8.99%), and *Caulobacter* (5.38%) (Figures 1A,B and Supplementary Figure 2). The alpha-diversity of the microbiota in ECB-M, indicated by the Shannon index, was significantly higher than that of ECB-D (*t* = 35.35, *P* < 0.0001; Figure 1C and Supplementary Table 2). The PCoA score plot showed an obvious separation between the two groups (PERMANOVA: *F* = 239.26, *R*^2^ = 0.95988, *P* < 0.003; Figure 1D).

Functional capacity analysis of the gut microbial communities revealed that 29 pathways enriched in each gut were similar among two groups and primarily associated with metabolism (Figure 1E). The ECB-associated bacterial symbionts contain genes involved in carbohydrate metabolism and amino acid metabolism. Genes involved in carbohydrate metabolism were significantly different between ECB-M and ECB-D (*t* = 30.13, *P* < 0.0001; Figure 1E and Supplementary Table 3). The results indicated that the host diet may alter the gut microbiota of ECB larvae and its associated functions.

### Cellulose Degrading Bacteria in the Larval Gut of European Corn Borer

**FIGURE 2:**
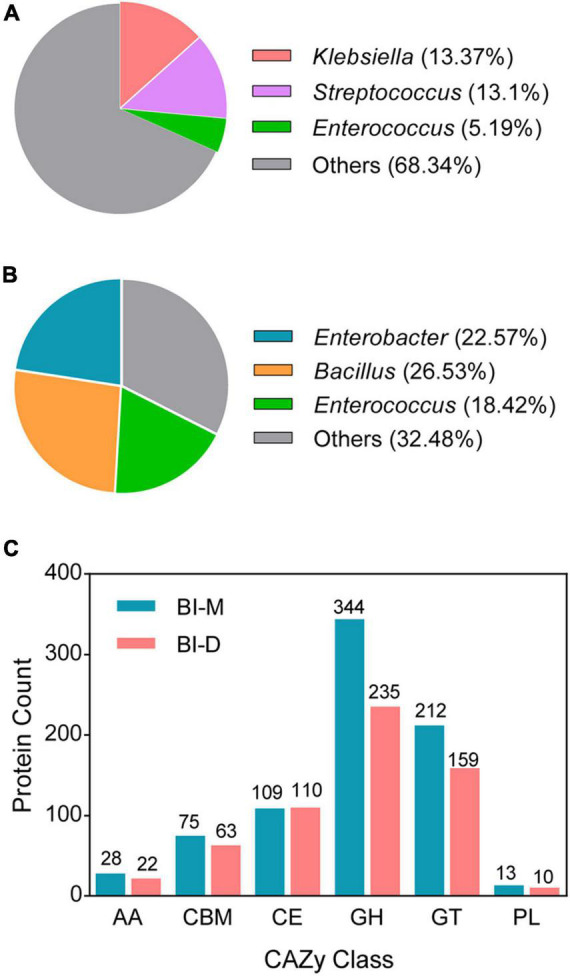
Draft genomes of two microbial consortia from the gut of ECB larvae fed with an artificial diet (BI-D) and maize plants (BI-M) respectively. Panel **(A)** and panel **(B)** represent the relative abundance of the dominant bacteria in BI-D and BI-M respectively. **(C)** The number of CAZyme genes defined in the draft genomes of BI-D and BI-M.

**FIGURE 3:**
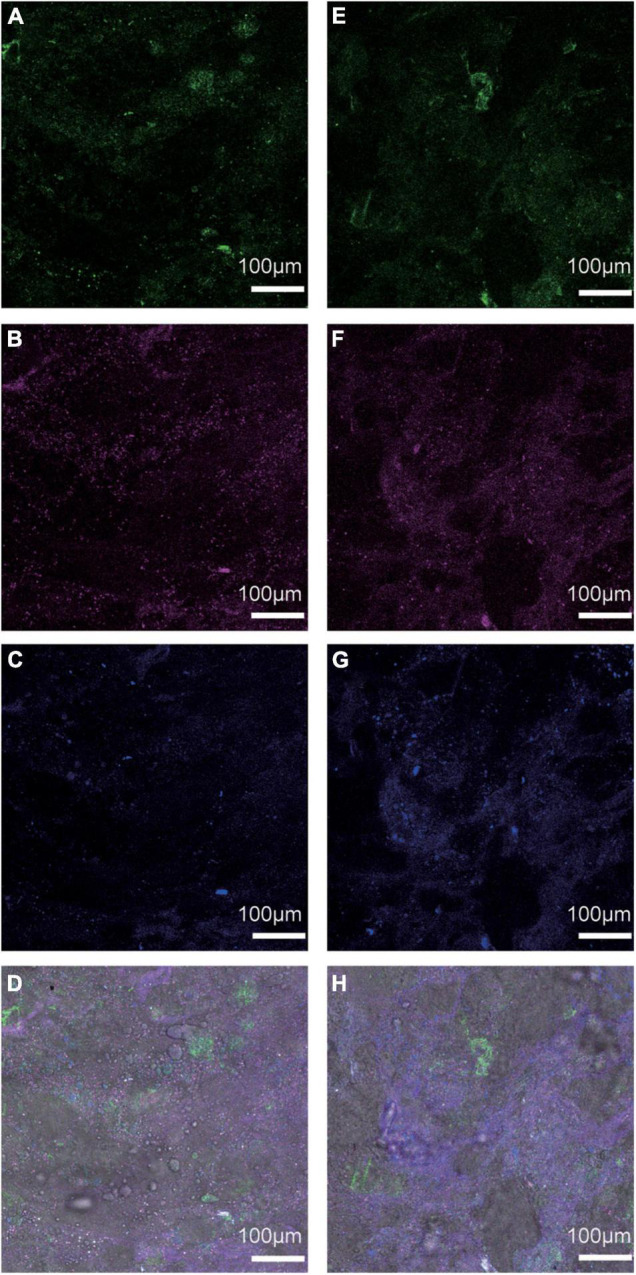
FISH analysis of the localization of **(A)** *Streptococcus* (green), **(B)** *Klebsiella* (purple), **(C)** *Enterococcus* (blue), and **(D)** three bacteria in the larval midgut of ECB-M, and **(E)** *Bacillus* (green), **(F)** *Enterobacter* (purple), **(G)** *Enterococcus* (blue), and **(H)** three bacteria in the larval midgut of ECB-D.

The two cellulolytic microbial consortia (BI-M and BI-D) produced clear zones around the colonies after Congo red staining (Supplementary Figure 1). The genomic features of the two bacterial consortia are as shown in Supplementary Table 4. Sequencing of the two consortia showed that *Klebsiella* (13.37%), *Streptococcus* (13.1%), and *Enterococcus* (5.19%) were the dominant genera in BI-M (Figure 2A and Supplementary Table 5), while *Bacillus* (26.53%), *Enterococcus* (18.42%), and *Enterobacter* (22.57%) were high in proportion in BI-D (Figure 2B and Supplementary Table 5). These bacterial genera were mainly located in the midgut of ECB larva (Figure 3).

A total of 781 CAZyme genes were detected from BI-M bacteria, which can be broken down into 344 GHs, 212 GTs, 75 CBMs, 109 CEs, 13 PLs, and 28 AAs. In comparison, 599 genes, including 235 GHs, 159 GTs, 63 CBMs, 110 CEs, 10 PLs, and 22 AAs were found in BI-D (Figure 2C and Supplementary Table 6). Within the most abundant CAZyme category (GHs), 48 families were identified in BI-M, mainly GH1 (β-glucosidase), GH2 (β-galactosidase, β-mannosidase, and β-glucuronidase), and GH13 (α-amylase, and α-glucosidase), whereas BI-D harboured 49 GHs, including GH18 (chitinase, and endo-β-N-acetylglucosaminidase) (Figure 2C and Table 1). These results suggested that bacterial consortia from the gut of larval ECB fed with artificial diets and maize exhibited variation in their GH repertoires.

**TABLE 1:** A partial list of CAZymes identified in the microbial consortia from the ECB larvae fed with an artificial diet (BI-D) or maize plants (BI-M)

	Number of proteins		
	
GHs	BI-M	BI-D	Known activities (http://www.cazy.org.)
GH1	22	39	β-glucosidase, β-galactosidase, β-mannosidase, others
GH2	6	6	β-galactosidase, β-mannosidase, β-glucuronidase, others
GH3	6	8	β-glucosidase, 1,4-β-xylosidase, 1,3-β-glucosidase, 1,4-β-glucosidase, others
GH4	9	16	α-glucosidase; α-galactosidase; α-glucuronidase, others
GH5	3	2	chitosanase, β-mannosidase, cellulase, 1,3-β-glucosidase, others
GH8	2	5	chitosanase, cellulase, licheninase, endo-1,4-β-xylanase, others
GH13	38	49	α-amylase, α-glucosidase, pullulanase, cyclomaltodextrinase, others
GH15	0	1	glucoamylase, glucodextranase, α,α-trehalase
GH16	0	3	endo-1,3-β-glucanase, licheninase, xyloglucanase, others
GH18	10	3	chitinase, endo-β-N-acetylglucosaminidase, others
GH19	1	1	chitinase
GH20	3	1	β-hexosaminidase, β-1,6-N-acetylglucosaminidase, others
GH23	12	20	lysozyme, peptidoglycan lyase
GH24	3	17	lysozyme
GH25	6	10	lysozyme
GH28	1	4	polygalacturonase, exo-polygalacturonase, others
GH31	5	5	α-glucosidase, α-1,3-glucosidase, α-xylosidase, α-glucan lyase, others
GH32	4	13	invertase, endo-inulinase, endo-levanase, others
GH33	2	0	sialidase, *trans*-sialidase, 2-keto-3-deoxynononic acid sialidase
GH35	2	1	β-galactosidase, exo-β-glucosaminidase
GH36	2	7	α-galactosidase, α-N-acetylgalactosaminidase, others
GH37	2	5	α,α-trehalase
GH38	6	5	α-mannosidase, α-1,3-1,6-mannosidase, others
GH39	0	2	α-L-iduronidase, β-xylosidase
GH42	0	1	β-galactosidase
GH43	4	13	β-xylosidase, β-1,3-xylosidase, xylanase, 1,3-β-galactosidase, others
GH51	3	4	α-L-arabinofuranosidase, endoglucanase
GH53	0	3	endo-β-1,4-galactanase
GH63	1	1	α-1,3-glucosidase, α-glucosidase
GH65	7	6	α,α-trehalase, maltose phosphorylase, trehalose phosphorylase, others
GH70	0	2	dextransucrase, alternansucrase, others
GH73	15	20	peptidoglycan hydrolase
GH74	3	0	endoglucanase, xyloglucanase
GH77	2	6	amylomaltase, 4-α-glucanotransferase
GH78	2	6	α-L-rhamnosidase
GH80	1	0	chitosanase
GH85	1	0	endo-β-N-acetylglucosaminidase
GH88	3	2	d-4,5 unsaturated β-glucuronyl hydrolase
GH92	2	3	α-1,2-mannosidase, α-1,3-mannosidase
GH94	1	2	cellobiose phosphorylase, cellodextrin phosphorylase, others
GH101	1	0	endo-α-N-acetylgalactosaminidase
GH102	1	2	peptidoglycan lytic transglycosylase
GH103	1	2	peptidoglycan lytic transglycosylase
GH104	0	4	peptidoglycan lytic transglycosylase
GH105	2	4	unsaturated rhamnogalacturonyl hydrolase
GH108	0	1	N-acetylmuramidase
GH109	26	29	α-N-acetylgalactosaminidase
GH112	1	0	lacto-N-biose phosphorylase, D-galactosyl-1,4-L-rhamnose phosphorylase
GH113	1	0	β-mannanase
GH114	1	1	endo-α-1,4-polygalactosaminidase
GH125	3	2	exo-α-1,6-mannosidase
GH126	2	1	amylase
GH127	0	3	L-arabinofuranosidase, 3-C-carboxy-5-deoxy-L-xylose hydrolase
GH129	1	0	N-acetylgalactosaminidase
GH136	1	0	lacto-N-biosidase
GH153	1	2	β-1,6-D-glucosamine hydrolase
GH154	2	1	β-glucuronidase
GH158	1	0	endo-β-1,3-glucanase

*GH, glycoside hydrolase.*

### Degradation Maize Cellulose by the Microbial Consortia *in vitro*

**FIGURE 4:**
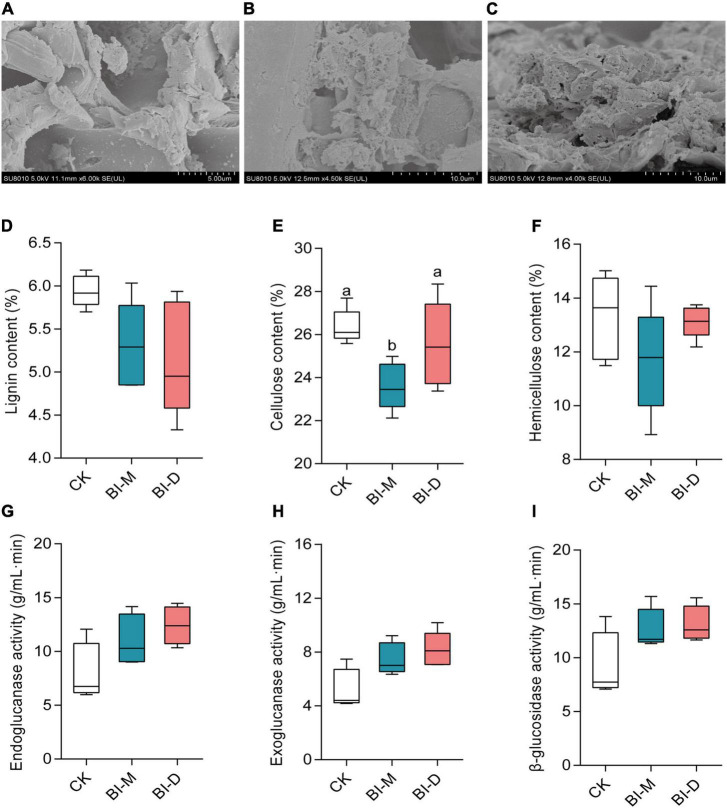
*In vitro* maize cellulose degradation by microbial consortia BI-D and BI-M. Scanning electron micrographs of **(A)** untreated maize particles, **(B)** maize particles treated by BI-D for 9 days, and **(C)** maize particles treated by BI-M for 9 days. The content of **(D)** lignin, **(E)** cellulose and **(F)** hemicellulose in maize particles after treated for 9 days. The cellulose-associated enzyme activity of **(G)** endoglucanase, **(H)** exoglucanase, and **(I)** β-glucosidase in the culture medium of maize particles treated with BI-D and BI-M for 9 days. Horizontal lines in the boxes represent group medians, and whiskers represent the 10th–90th percentiles. Superscripts (a, b) indicate significant differences between different groups (*P* < 0.05).

Scanning electron microscopy analysis showed that the surfaces of the untreated maize particles were smooth (Figure 4A), whereas the structure of maize particles treated with both BI-D and BI-M was destroyed (Figures 4B,C). Cellulose content in maize particles treated with BI-M was significantly lower than those treated with BI-D or the control (*Kruskal–Wallis* test: *X*^2^ = 6.72, df = 2; *P* = 0.0259; Figure 4E). Meanwhile, lignin, cellulose, and hemicellulose were all reduced on maize particles treated with BI-D and BI-M compared to untreated ones (Figures 4D–F). The cellulolytic activity of endoglucanase, β-glucosidase, and exoglucanase in the cultures of maize particles treated with BI-D and BI-M were higher than those in untreated maize particles, albeit not significantly (Figures 4G–I). Taken together, the results suggested that the bacterial consortia BI-D and BI-M exhibited the ability of maize cellulose degradation *in vitro* to different extents.

### Metabolomic Profiles of Maize Cellulose *in vitro*

**FIGURE 5:**
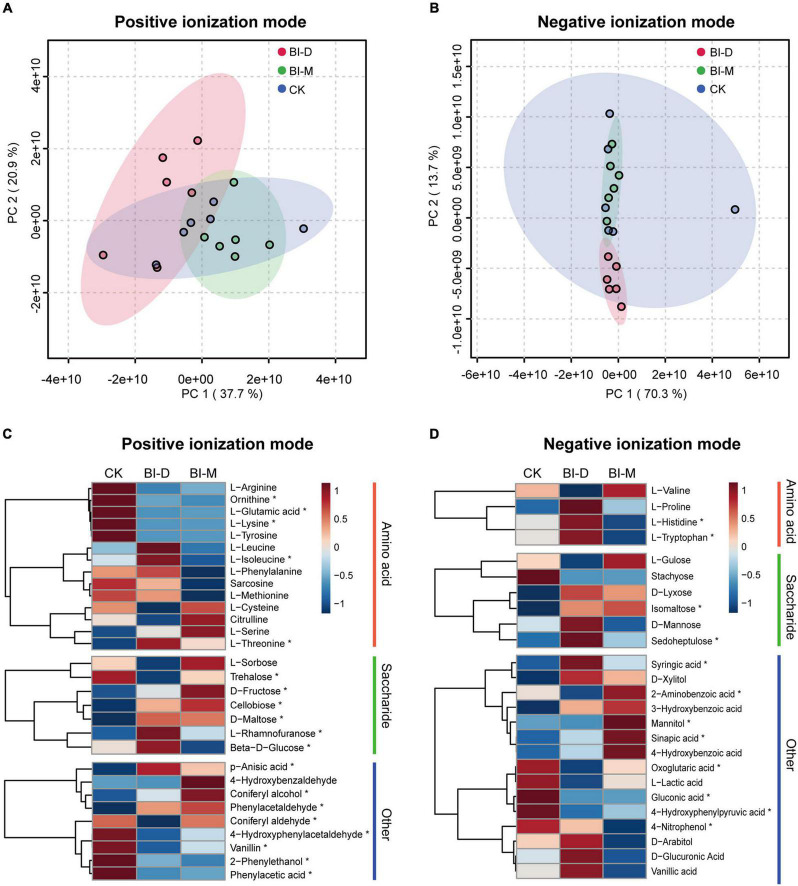
Metabolomic analysis of *in vitro* degradation of maize particles by two microbial consortia. PCA score plot of metabolic profiles in **(A)** positive and **(B)** negative ionization modes. Colored circles represent the metabolic profiles of individual samples. Ellipses indicate 95% confidence region for each group. Relative metabolite levels of maize cellulolytic degradation by two bacterial isolates in **(C)** positive and **(D)** negative ionization modes. The color scale shows levels for each metabolite relative to the average abundance. Asterisks indicate significant differences (*P* < 0.05) between each group. Summary statistics are provided in Supplementary Table 7.

Untargeted metabolome analyses document that maize particles treated by two microbial consortia generate distinctive metabolomic profiles, with enrichment for specific monosaccharides and amino acids (Figure 5). The principal component analysis (PCA) shows the difference in metabolic profiles of maize treated by the consortia (Figures 5A,B). The major axes of the PCoA explain 58.6% of the variance for positive ionization mode in metabolomic profiles (PC1 = 37.7% and PC2 = 20.9%; Figure 5A), and 84.0% of the variance (PC1 = 70.3% and PC2 = 13.7%) for negative ionization mode (Figure 5B).

For both ionization modes, a comparison of relative metabolite levels with a one-way ANOVA showed that 38.9% (210 of 540) of the monitored metabolites varied significantly (*P* < 0.05) among three samples (Supplementary Table 7). The levels of D-fructose, cellobiose, D-maltose, L-rhamnofuranose and isomaltose in maize particles treated with both BI-D and BI-M were significantly higher than those in untreated maize particles (D-fructose: *F* = 10.146, *P* < 0.01; cellobiose: *F* = 19.358, *P* < 0.001, D-maltose: *F* = 9.95, *P* < 0.01; L-rhamnofuranose: *F* = 7.931, *P* < 0.01, and isomaltose: *F* = 10.702, *P* < 0.01; Figures 5C,D and Supplementary Table 7). Additionally, there were significant differences in levels of several amino acids among the metabolic profiles of maize particles treated or untreated by bacterial isolates, including ornithine (*F* = 23.38, *P* < 0.001), L-glutamic acid (*F* = 13.455, *P* < 0.001), L-lysine (*F* = 5.21, *P* < 0.05), L-isoleucine (*F* = 9.4291, *P* < 0.01), L-threonine (*F* = 17.93, *P* < 0.001), L-histidine (*F* = 40.595, *P* < 0.0001), and L-tryptophan (*F* = 43.127, *P* < 0.0001) (Figures 5C,D and Supplementary Table 7).

Several small molecular aromatic metabolites, such as phenol, 2-phenylethanol, coniferyl alcohol, 4-hydroxybenzaldehyde, 4-hydroxyphenylacetaldehyde, p-anisic acid, vanillin, phenylacetic acid, phenylacetaldehyde, 2-aminobenzoic acid, mannitol, 4-nitrophenol, 4-hydroxyphenylpyruvic acid, syringic acid, sinapic acid, sinapyl alcohol, D-glucuronic acid, vanillic acid, D-arabitol, 3-hydroxybenzoic acid, D-xylitol, and L-lactic acid, were detected in the samples treated by both microbial consortia (Figures 5C,D and Supplementary Table 7).

## Discussion

**FIGURE 6:**
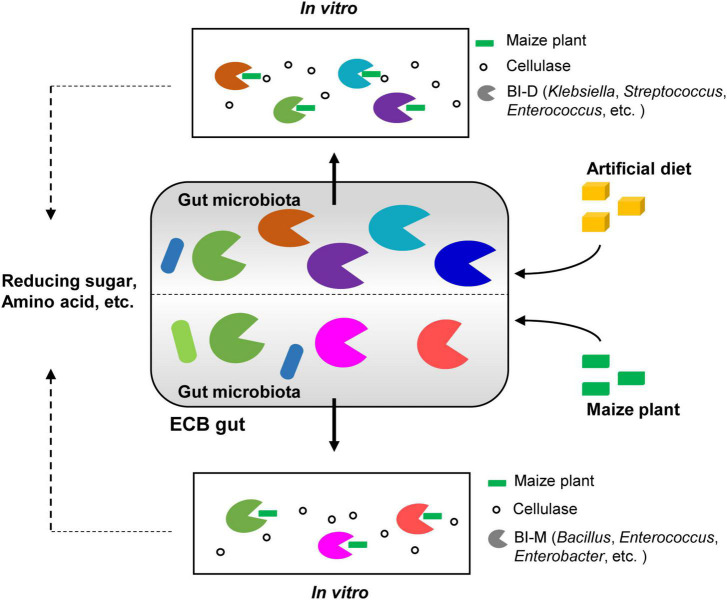
Graphical summary of the main results. Diet shapes the gut bacterial community of ECB larvae. Two bacterial isolates from the guts of ECB larvae exhibited the ability to degrade maize cellulose to varying degrees *in vitro* and produced distinctive metabolomic profiles, including reduced sugars and amino acids.

While most insects rely on gut bacteria to digest cellulose and produce sugars that are then available to the host, this has been disputed in Lepidopteran larvae due to their simple gut morphology and rapid digestive throughput. In this study, we demonstrate that the composition of gut microbiota in *Ostrinia nubilalis* is determined by its diet. Draft genome and metabolome analyses showed that two cellulolytic microbial consortia from hosts feeding on different diets have different capabilities to digest maize lignocellulose *in vitro* and release different downstream products. SEM provides direct evidence for the degradation of maize cellulose by the ECB gut microbial consortia *in vitro* (Figure 6). A thorough understanding of how cellulosic biomass is digested by gut microbiomes would not only solidify the importance of the gut microbiota in the ecology and evolution of insects, but also provide valuable insights toward the industrial application of microbiota for cellulose conversion.

### Diet Shapes Gut Microbiota of the *Ostrinia nubilalis* Larva

Diet is the primary determinant of bacterial community structures in the gut of many herbivorous lepidopteran larvaes (Broderick et al., 2004; Robinson et al., 2010; Mason et al., 2020). Our findings that Firmicutes are the dominant phylum in ECB fed on an artificial diet were similar to that of a previous study (Belda et al., 2011), suggesting that the dominant phyla are relatively stable in the guts of hosts on the same diet. We also show that the dominant phyla in the gut microbiota of ECB fed on maize are distinct from bacteria in ECB reared on pepper tissues (Belda et al., 2011). These observations indicated that diet influences the proliferation and stability of gut microbiota in *O. nubilalis* larvae (Belda et al., 2011). In addition, we found that the diversity of gut microbiota in ECB fed on maize was significantly higher than that in ECB fed an artificial diet, and included taxa widespread in other environments, such as *Pseudomonas* and *Bacillus*. These bacteria might have been actively or passively acquired from microbial communities inhabiting plant surfaces (Partida-Martinez and Heil, 2011). This indicates that these bacteria can establish in the host gut and are not merely transient associates. Another noteworthy observation was that the ECB larva fed with an artificial diet harbored a rather simple gut microbiota consisting mostly of genus belonging to *Enterococcus* (96.89%). *Enterococcus* species, which exhibit lignocellulose degradation activity and are commonly present in gut symbiotic microbiota of insects, were also found in the rumen of ruminants (Nyonyo et al., 2014; Shil et al., 2014; Luo et al., 2019; Przemieniecki et al., 2020). The existence of this species in ECB-D provided an important basis for their high cellulolytic ability. Overall, our findings raise the possibility that diet may exert a strong selection pressure and shape the composition of microbiota harboured in the insect intestinal tract. Although it has been speculated that microbes cannot persist in the gut of herbivorous Lepidopteran larvae or contribute to feeding and digestion (Hammer et al., 2017), here we show that *O. nubilalis*-associated bacteria serve various functions for their host. Thus, it is likely that the degree of reliance on microbes of *O. nubilalis* is underappreciated.

### The Diversity of Cellulolytic Bacteria in the Gut of *Ostrinia nubilalis* Larvae

Insect gut microbiomes are considered as an endless reservoir for the construction of synthetic microbial consortia for biomass utilization and identification of enzymes of industrial importance (Marynowska et al., 2020). The microbial consortia have more balanced cellulolytic enzyme complements than individual strains, tolerance changes in environmental conditions, and that they have high functional redundancy (Ali et al., 2020). Most dominant genera (*Enterococcus*, *Enterobacter*, and *Streptococcus*) in both microbial consortia obtained from the guts of ECB larvae have been shown to exhibit cellulolytic properties and produce enzymes that efficiently metabolize cellulosic biomass in other host species (Schmid et al., 2014; Manfredi et al., 2015). *Bacillus* species, which exhibit lignocellulose degradation activity and are known to be safe strains for cellulase production (Ma et al., 2015), were also found in the gut of *Holotrichia parallela* (Huang et al., 2012), the wood-feeding termite (Tsegaye et al., 2018), and *Cyrtotrachelus buqueti* (Li et al., 2020). However, previous studies of Lepidopteran larvae have typically observed cellulolytic bacteria from different taxa. For example, the strain *Klebsiella* sp. MD21 has been found responsible for cellulose digestion in the gut of the cotton bollworm (*Helicoverpa armigera*) (Dar et al., 2018). In particular, a previous study also focusing on *O. nubilalis* suggests that *Micrococcus* and *Acinetobacter* are the main groups with cellulolytic properties (Vilanova et al., 2012), contrasting our findings. This can be interpreted as host genotype specificity.

Additionally, FISH analysis of the bacteria isolates showed that bacteria with cellulolytic capabilities were mainly located in the midgut of *O. nubilalis*, unlike other insect species where they are typically found in the hindgut (Warnecke et al., 2007; Mikaelyan et al., 2014). Knowing that differences in gut structure can lead to major functional differences in insects (Engel and Moran, 2013; Ni and Tokuda, 2013; Brune and Dietrich, 2015), our results suggest that the midgut of ECB is where digestion of maize cellulose occurs, although its occurrence *in vivo* is still undetermined.

### The Mechanism of Cellulose Degradation by Microbial Consortia

The presence and diversity of CAZymes in the functional categories of cellulase, hemicellulase, or pectinase/esterase were assessed using records from the CAZy Database. The gut bacteria of insects produce a diverse repertoire of CAZymes (Lombard et al., 2014). The class with the most genes detected in both consortia, GH, is enzymes that play a key role in carbohydrate degradation by acting on glycosidic bonds (Li et al., 2020). Many of the major GH families found in BI-D and BI-M have been previously documented to be abundant in lignocellulolytic microbial consortia, such as those grown on wheat straw, xylan, and xylose (Jiménez et al., 2015). The GH families encompass a variety of hydrolytic enzymes, e.g., cellulases, endo- and exo-glucanases, arabinofuranosidases, endoxylanases, cellobiohydrolases, and xyloglucanases, thus, GHs might be responsible for the degradation of maize cellulose *in vitro.* Specifically, members of GH1 are often potential β-glucosidases that utilize cellobiose, and the GH43s are considered as another important member for the degradation of hemicelluloses (Romero et al., 2012). The GH3 family is also an important component of cellulose degradation systems and might have enhanced the ability to degrade cellulose in both consortia. Additionally, CEs, PLs, and AAs also contribute to the decomposition of lignocellulose. For example, the major CEs and AAs families found in two microbial consortia (BI-D and BI-M) were CE1, CE4, CE10, AA4, and AA7, all associated with cellulolytic functions (Mamo et al., 2006; Zhang et al., 2011; Li et al., 2020). These cellulolytic enzymes can also act synergistically and increase the overall efficiency of substrate utilization (Murashima et al., 2003). The high diversity of CAZyme families in BI-D and BI-M may have made them potent cellulose decomposers, and the diversity of CAZyme families in BI-M strongly suggests that it is a promising consortium to facilitate cellulose degradation.

Additionally, digestive efficiency *in vitro* differs among bacteria. It can also depend on the metabolic priorities of the bacteria, cellulase activity, and environmental factors such as temperature and pH (Watanabe and Tokuda, 2010; Engel and Moran, 2013). In our results, the bacteria isolates exhibited relatively low digestive efficiency of maize *in vitro*; it is unclear which of the above hypotheses can explain this phenomenon. The structure and underlying mechanisms of gut bacteria associated CAZymes and the extent of these roles await further investigation.

### Variation in the Metabolome Among Degradation Products of Maize

Products of lignocellulose degradation include many monosaccharides, aromatic compounds, and others (Caffall and Mohnen, 2009; Watanabe and Tokuda, 2010). Here, we find that reduced sugars such as D-fructose, cellobiose, D-maltose, L-rhamnofuranose, and, isomaltose accumulated in maize particles treated by the two microbial consortia compared to the control. This may be due to the larger microbial diversity and higher CAZyme-encoding gene numbers in microbial consortia. GHs acts on glycosidic bonds in crystalline polysaccharides, amino polysaccharides, and other complex polysaccharides present in the biomass and crystalline polysaccharides, amino polysaccharides, and other complex polysaccharides present in the biomass and transform them into fermentable sugars (Alonso-Pernas et al., 2017; Sethupathy et al., 2021). In contrast, the contents of glucose and fructose decreased. Therefore, it is possible that the bacteria absorbed some of the released sugars.

Additionally, gut bacteria often provide amino acids to their host (Moran and Baumann, 2000; Powell et al., 2016). In this study, we find that the levels of many amino acids (e.g., ornithine, L-glutamic acid, L-lysine, L-isoleucine, L-threonine, L-histidine, and L-tryptophan) increase in the metabolic profiles of maize particles treated with bacteria compared to those that were untreated, suggesting that gut bacteria can produce not only sugars but also amino acids *in vitro*. However, due to differences in physicochemical conditions of the gut compartments such as pH, redox potential, and substrates, it remains to be experimentally validated whether gut bacteria can provide amino acids to benefit itself or its host *in vivo*.

### Application Potential of Gut Microbiota of European Corn Borer in Cellulose Bioconversion

The potential application of insect gut microbiota in cellulose degradation can alleviate negative environmental impacts in current methods (Stephen et al., 2012; Xie et al., 2019). However, the industrialization of cellulolytic microbiota remains a challenge due to its low efficiency and high cost (Sun and Scharf, 2010). Here, we establish that microbial consortia from ECB are capable of degrading maize cellulose with relatively low efficiency. This potential could be further extended through genetic engineering for efficiency. Screening and developing native cellulolytic microbial consortium from the gut microbiota and applying them to lignocellulosic biomass based biofuel production is of great significance in accelerating bioconversion of lignocellulose-rich wastes.

## Conclusion

This work shows that diet shapes the gut microbial composition in *O. nubilalis*. The two cellulolytic microbial consortia, mainly belonging to Firmicutes or Proteobacteria, exhibit the ability to degrade maize cellulose *in vitro*. These results offer valuable microbiota resources for lignocellulose bioconversion and have significant potential in industrial applications. Future research will analyze the enzymatic properties of novel bacteria or genes to clarify the mechanisms of cellulose digestion in insects.

## Data Availability Statement

The datasets presented in this study can be found in online repositories. The names of the repository/repositories and accession number(s) can be found below: NCBI, PRJNA636935.

## Author Contributions

JL and TS conceived and designed the experiments. JL and JZ performed the experiments. JZ, ZD, and SW analyzed the data. JL and TS wrote the manuscript. All authors revised the manuscript and approved it for publication.

## Conflict of Interest

The authors declare that the research was conducted in the absence of any commercial or financial relationships that could be construed as a potential conflict of interest.

## Publisher’s Note

All claims expressed in this article are solely those of the authors and do not necessarily represent those of their affiliated organizations, or those of the publisher, the editors and the reviewers. Any product that may be evaluated in this article, or claim that may be made by its manufacturer, is not guaranteed or endorsed by the publisher.

## Footnotes

1
http://drive5.com/uparse/

2
https://www.microbiomeanalyst.ca/

3
http://www.usadellab.org/cms/index.php?page=trimmomatic

4
http://bcb.unl.edu/dbCAN2/blast.php
