# Supplementary material for: Gut Microbiota of Ostrinia nubilalis Larvae Degrade Maize Cellulose
Source: Front Microbiol. 2022 Apr 11;13:816954. doi: 10.3389/fmicb.2022.816954 (PMC9039043; doi:10.3389/fmicb.2022.816954)
Supplement: Supplementary file 1 [file Data_Sheet_1.docx]

Supplementary Material

#
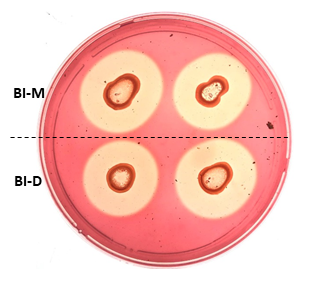
Supplementary Figures

**Figure S1**. Screening of cellulolytic microbial consortia by covering the Petri dishes with Congo red dye. A clear zone surrounding a colony is indicative of carboxymethyl cellulose (CMC) hydrolysis by secreted CMCase.

# Supplementary Figures


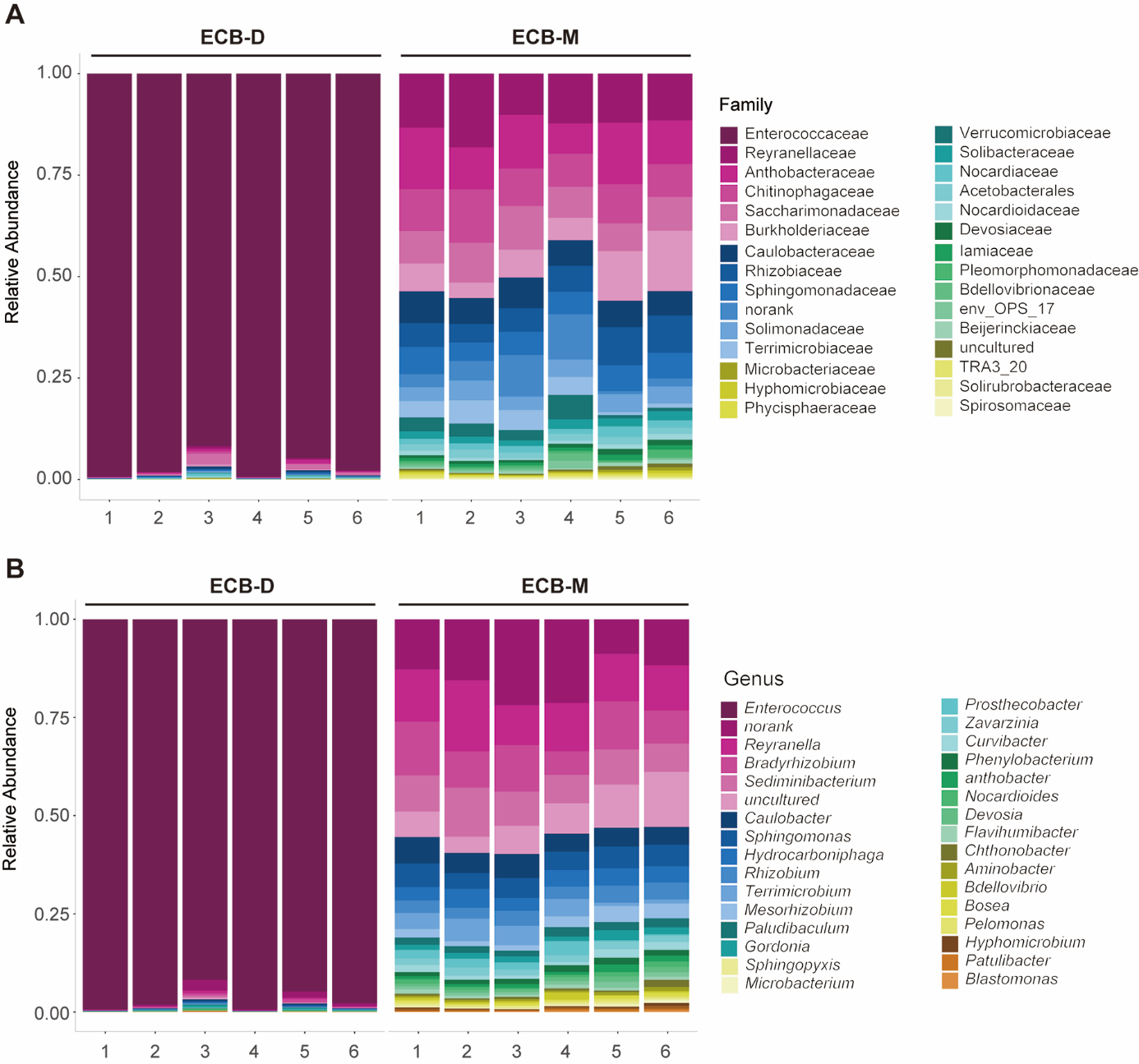


**Figure S2**. Relative abundance of microbiota in the ECB larvae fed with an artificial diet (ECB-D) or maize plants (ECB-M) at the family (A) and genus (B) level.

# Supplementary Tables

**Table S1**. Primers and probes used for localization (FISH) of bacterial symbionts in the gut of ECB.

| Primer type | Primer name | Target | Primer sequence (5’-3’) | 5’ mod | Reference |
| --- | --- | --- | --- | --- | --- |
| FISH | EUB338 | *Bacillus* | GCTGCCTCCCGTAGGAGT | FITC | Amann *et al*., 1990 |
|  | StrepF/492 | *Streptococcus* | GTTAGCCGTCCCTTTCTGG | FITC | Franks *et al.*, 1998 |
|  | Kle834 | *Klebsiella* | TTGTTCCCTTGAGGAGTGGCTTC | CY3 | Ootsubo *et al.*, 2002 |
|  | ENC 221 | *Enterobacter* | CACCGCGGGTCCATCCATCA | CY3 | Wellinghausen *et al.*, 2007 |
|  | Ecf459 | *Enterococcus* | GGGATGAACATTTTACTC | CY5 | Behr *et al.,* 2000 |

**Table S2**. Alpha diversity of bacterial communities of the gut of ECB larvae fed with maize plant (ECB-M) or artificial diets (ECB-D).

|  | ECB-M | ECB-D | *P* (Mann-Whitney U) |
| --- | --- | --- | --- |
| Good’s coverage | 100.00 ± 0.001 | 99.98 ± 0.004 | 0.002 (0) |
| Observed OTUs | 50.50 ± 0.342 | 42.67 ± 3.670 | 0.067 (6.5) |
| Chao 1 | 50.50 ± 0.340 | 46.64 ± 3.080 | 0.744 (15.5) |
| Inverse Simpson | 1.06 ± 0.003 | 4.90 ± 0.393 | 0.002 (0) |
| Fisher | 5.97 ± 0.042 | 4.91 ± 0.472 | 0.026 (4) |

The data shows mean ± SEM.

**Table S3**. Main function analysis of the microbiomes presents in the gut of ECB larvae fed with maize plant (ECB-M) or artificial diets (ECB-D).

| KEGG category | Ko abundance (Mean ± SEM) | | Significant |
| --- | --- | --- | --- |
|  | ECB-M | ECB-D |  |
| Cell Growth and Death | 0.0074 ± 0.00007 | 0.00481 ± 0.00008 | *** |
| Cell Motility | 0.03021 ± 0.0005 | 0.00879 ± 0.00064 | *** |
| Transport and Catabolism | 0.00363 ± 0.00005 | 0.00156 ± 0.00008 | *** |
| Membrane Transport | 0.12902 ± 0.00256 | 0.17371 ± 0.00229 | *** |
| Signal Transduction | 0.02211 ± 0.00007 | 0.01696 ± 0.00011 | *** |
| Signaling Molecules and Interaction | 0.00192 ± 0.00002 | 0.00206 ± 0.00001 | ** |
| Folding, Sorting and Degradation | 0.02061 ± 0.0004 | 0.01957 ± 0.00007 | ns |
| Replication and Repair | 0.06062 ± 0.00081 | 0.08324 ± 0.00068 | *** |
| Transcription | 0.02107 ± 0.00008 | 0.03084 ± 0.00037 | *** |
| Translation | 0.03624 ± 0.00064 | 0.05557 ± 0.00056 | *** |
| Amino Acid Metabolism | 0.10776 ± 0.00068 | 0.07865 ± 0.00128 | *** |
| Biosynthesis of Other Secondary Metabolites | 0.00953 ± 0.0001 | 0.00696 ± 0.00009 | *** |
| Carbohydrate Metabolism | 0.10131 ± 0.00039 | 0.13599 ± 0.00108 | *** |
| Energy Metabolism | 0.05691 ± 0.00116 | 0.04745 ± 0.00035 | *** |
| Enzyme Families | 0.01719 ± 0.00035 | 0.0175 ± 0.00004 | ns |
| Glycan Biosynthesis and Metabolism | 0.01761 ± 0.00051 | 0.01741 ± 0.00005 | ns |
| Lipid Metabolism | 0.0404 ± 0.00035 | 0.03061 ± 0.00049 | *** |
| Metabolism of Cofactors and Vitamins | 0.04018 ± 0.00059 | 0.02729 ± 0.0005 | *** |
| Metabolism of Other Amino Acids | 0.02194 ± 0.00026 | 0.01582 ± 0.00023 | *** |
| Metabolism of Terpenoids and Polyketides | 0.02266 ± 0.00013 | 0.01833 ± 0.00026 | *** |
| Nucleotide Metabolism | 0.02902 ± 0.00026 | 0.04288 ± 0.00044 | *** |
| Xenobiotics Biodegradation and Metabolism | 0.04887 ± 0.00144 | 0.02343 ± 0.00111 | *** |
| Circulatory System | 0.0005 ± 0.00002 | 0.00005 ± 0.00001 | *** |
| Digestive System | 0.00035 ± 0.00002 | 0.0001 ± 0.000001 | *** |
| Endocrine System | 0.00393 ± 0.00004 | 0.00134 ± 0.00012 | *** |
| Environmental Adaptation | 0.00141 ± 0.00001 | 0.00099 ± 0.00001 | *** |
| Excretory System | 0.00031 ± 0.00001 | 0.00003 ± 0.00001 | *** |
| Immune System | 0.00041 ± 0.00001 | 0.00004 ± 0.00001 | *** |
| Nervous System | 0.00093 ± 0.00001 | 0.00047 ± 0.00002 | *** |
| Others | 0.14595 ± 0.00106 | 0.13758 ± 0.00009 | *** |

Abbreviation: ns, no significant.

**Table S4**. General draft whole-genome features of two microbial consortia from the gut of ECB larvae fed with an artificial diet (BI-D) or maize plant (BI-M).

| Main terms | Characteristic | BI-M | BI-D |
| --- | --- | --- | --- |
| Sequenced data | Raw data (Mb) | 7374.13 | 4791.18 |
|  | Clean data (Mb) | 6218.95 | 4140.42 |
|  | GC content (%) | 46.06 | 47.13 |
|  | Q20 (%) | 98.29 | 98.34 |
|  | Q30 (%) | 93.96 | 94.1 |
| Assembly results | Total scaffolds | 3542 | 233 |
|  | Total base | 23007658 | 17956735 |
|  | No. of large scaffolds (>1 kbp) | 2457 | 203 |
|  | Bases in large scaffolds | 22210022 | 17935677 |
|  | Largest length | 842167 | 3523460 |
|  | Scaffold N50 | 30907 | 230637 |
|  | Scaffold N90 | 3009 | 51820 |
|  | G+C content (%) | 46.27 | 45.53 |
|  | N rate (%) | 0.6 | 0 |
| Predicted genes | Gene Number | 25459 | 17854 |
|  | Gene Total Length (bp) | 20189322 | 15823908 |
|  | Gene Average Length (bp) | 793 | 886 |
|  | Gene's GC Content (%) | 47.2 | 46.5 |
|  | Gene/Genome (%) | 87.8 | 88.1 |
| ncRNA analysis | tRNA | 350 | 303 |
|  | tRNA | 350 | 303 |
|  | 5S | 18 | 24 |
|  | 16S | 4 | 3 |
|  | 23S | 4 | 4 |
| Gene function annotation | Total protein (#) | 25459 | 17854 |
|  | NR | 24941 | 17431 |
|  | GO | 17402 | 11616 |
|  | eggNOG | 19850 | 14201 |
|  | KEGG | 13662 | 9566 |
|  | Swiss-Prot | 17663 | 11827 |

**Table S5**. The compositions of two microbial consortia from the gut of ECB larvae fed with an artificial diet (BI-D) or maize plants (BI-M) based on draft genome data.

| Classification | Number of sequence (% of assembly) | |
| --- | --- | --- |
|  | BI-M | BI-D |
| Bacteria | 18879671 (90.73%) | 13397106 (96.7%) |
| Proteobacteria | 10678722 (51.32%) | 6406117 (46.245) |
| Gammaproteobacteria | 10579055 (50.84%) | 6350739 (45.84%) |
| Enterobacterales | 10430041 (50.13%) | 6262742 (45.21%) |
| Enterobacteriaceae | 9850855 (47.34%) | 5861862 (42.31%) |
| *Klebsiella* | **2782458 (13.37%)** | 5951 (0.04%) |
| *Enterobacter* | 119448 (0.57%) | **3126305 (22.57%)** |
| Firmicutes | 7869346 (37.82%) | 6410178 (46.27%) |
| Bacilli | 7810254 (37.54%) | 6380646 (46.06%) |
| Lactobacillales | 3904276 (18.76%) | 2613954 (18.87%) |
| Streptococcaceae | 2725385 (13.1%) | 8827 (0.06%) |
| *Streptococcus* | **2688276 (12.92%)** | 1707 (0.01%) |
| Enterococcaceae | 1090426 (5.24%) | 2563216 (18.5%) |
| *Enterococcus* | **1080003 (5.19%)** | **2551879 (18.42%)** |
| Bacillales | 3835651 (18.43%) | 3724514 (26.88%) |
| Bacillaceae | 98911 (0.48%) | 3686719 (26.61%) |
| *Bacillus* | 86128 (0.41%) | **3675828 (26.53%)** |

**Table S6**. Summary analysis of the number of CAZyme genes defined in the draft genomes of two microbial consortia BI-D and BI-M.

| CAZy family | Class name | Number of genes detected | | Known activities (http://www.cazy.org.) |
| --- | --- | --- | --- | --- |
|  |  | BI-M | BI-D |  |
| AA | AA2 | 5 | 1 | lignin peroxidase, peroxidase, others |
|  | AA3 | 7 | 5 | cellobiose dehydrogenase, glucose 1-oxidase, pyranose oxidase, others |
|  | AA4 | 2 | 2 | vanillyl-alcohol oxidase |
|  | AA6 | 11 | 7 | 1,4-benzoquinone reductase |
|  | AA7 | 1 | 2 | glucooligosaccharide oxidase, chitooligosaccharide oxidase |
|  | AA10 | 2 | 5 | chitin, cellulose |
| CE | CE1 | 37 | 40 | acetyl xylan esterase, cinnamoyl esterase, carboxylesterase, others |
|  | CE10 | 24 | 18 | arylesterase, carboxyl esterase, acetylcholinesterase, others |
|  | CE11 | 2 | 1 | UDP-3-0-acyl N-acetylglucosamine deacetylase |
|  | CE12 | 4 | 3 | pectin acetylesterase, rhamnogalacturonan acetylesterase, acetyl xylan esterase |
|  | CE14 | 3 | 3 | diacetylchitobiose deacetylase, others |
|  | CE15 | 1 | 1 | 4-O-methyl-glucuronoyl methylesterase |
|  | CE3 | 7 | 7 | acetyl xylan esterase |
|  | CE4 | 16 | 24 | acetyl xylan esterase, chitin deacetylase, chitooligosaccharide deacetylase, others |
|  | CE5 | 0 | 2 | acetyl xylan esterase, cutinase |
|  | CE6 | 1 | 0 | acetyl xylan esterase |
|  | CE7 | 4 | 3 | acetyl xylan esterase, cephalosporin-C deacetylase |
|  | CE8 | 3 | 2 | pectin methylesterase |
|  | CE9 | 7 | 6 | N-acetylglucosamine 6-phosphate deacetylase, N-acetylgalactosamine-6-phosphate deacetylase |
| GH | GH1 | 22 | 39 | β-glucosidase, β-galactosidase, β-mannosidase, others |
|  | GH2 | 6 | 6 | β-galactosidase, β-mannosidase, β-glucuronidase, others |
|  | GH3 | 6 | 8 | β-glucosidase, 1,4-β-xylosidase, 1,3-β-glucosidase, 1,4-β-glucosidase, others |
|  | GH4 | 9 | 16 | α-glucosidase; α-galactosidase; α-glucuronidase, others |
|  | GH5 | 3 | 2 | chitosanase, β-mannosidase, Cellulase, 1,3-β-glucosidase β-1,4-cellobiosidase, others |
|  | GH8 | 2 | 5 | chitosanase, cellulase, licheninase, endo-1,4-β-xylanase, others |
|  | GH13 | 38 | 49 | α-amylase, a-glucosidase, pullulanase, cyclomaltodextrinase, others |
|  | GH15 | 0 | 1 | glucoamylase, glucodextranase, α,α-trehalase |
|  | GH16 | 0 | 3 | endo-1,3-β-glucanase (EC 3.2.1.39), endo-1,3(4)-β-glucanase, xyloglucanase, others |
|  | GH18 | 10 | 3 | chitinase, endo-β-N-acetylglucosaminidase, others |
|  | GH19 | 1 | 1 | chitinase |
|  | GH20 | 3 | 1 | β-hexosaminidase, β-1,6-N-acetylglucosaminidase, others |
|  | GH23 | 12 | 20 | lysozyme, peptidoglycan lyase |
|  | GH24 | 3 | 17 | lysozyme |
|  | GH25 | 6 | 10 | lysozyme |
|  | GH28 | 1 | 4 | polygalacturonase, exo-polygalacturonase, exo-polygalacturonosidase, others |
|  | GH31 | 5 | 5 | α-glucosidase, α-1,3-glucosidase, α-xylosidase, α-glucan lyase, others |
|  | GH32 | 4 | 13 | invertase, endo-inulinase, endo-levanase, others |
|  | GH33 | 2 | 0 | sialidase, trans-sialidase, 2-keto-3-deoxynononic acid sialidase |
|  | GH35 | 2 | 1 | β-galactosidase, exo-β-glucosaminidase |
|  | GH36 | 2 | 7 | α-galactosidase, α-N-acetylgalactosaminidase, others |
|  | GH37 | 2 | 5 | α,α-trehalase |
|  | GH38 | 6 | 5 | α-mannosidase, α-1,3-1,6-mannosidase, others |
|  | GH39 | 0 | 2 | α-L-iduronidase, β-xylosidase |
|  | GH42 | 0 | 1 | β-galactosidase |
|  | GH43 | 4 | 13 | β-xylosidase, β-1,3-xylosidase, α-L-arabinofuranosidase, xylanase, 1,3-β-galactosidase, |
|  | GH51 | 3 | 4 | α-L-arabinofuranosidase, endoglucanase |
|  | GH53 | 0 | 3 | endo-β-1,4-galactanase |
|  | GH63 | 1 | 1 | α-1,3-glucosidase, α-glucosidase |
|  | GH65 | 7 | 6 | α,α-trehalase, maltose phosphorylase, trehalose phosphorylase, others |
|  | GH70 | 0 | 2 | dextransucrase, alternansucrase, others |
|  | GH73 | 15 | 20 | peptidoglycan hydrolase |
|  | GH74 | 3 | 0 | endoglucanase, xyloglucanase |
|  | GH77 | 2 | 6 | amylomaltase, 4-α-glucanotransferase |
|  | GH78 | 2 | 6 | α-L-rhamnosidase |
|  | GH80 | 1 | 0 | chitosanase |
|  | GH85 | 1 | 0 | endo-β-N-acetylglucosaminidase |
|  | GH88 | 3 | 2 | d-4,5 unsaturated β-glucuronyl hydrolase |
|  | GH92 | 2 | 3 | mannosyl-oligosaccharide α-1,2-mannosidase, mannosyl-oligosaccharide α-1,3-mannosidase |
|  | GH94 | 1 | 2 | cellobiose phosphorylase, cellodextrin phosphorylase, others |
|  | GH101 | 1 | 0 | endo-α-N-acetylgalactosaminidase |
|  | GH102 | 1 | 2 | peptidoglycan lytic transglycosylase |
|  | GH103 | 1 | 2 | peptidoglycan lytic transglycosylase |
|  | GH104 | 0 | 4 | peptidoglycan lytic transglycosylase |
|  | GH105 | 2 | 4 | unsaturated rhamnogalacturonyl hydrolase |
|  | GH108 | 0 | 1 | N-acetylmuramidase |
|  | GH109 | 26 | 29 | α-N-acetylgalactosaminidase |
|  | GH112 | 1 | 0 | lacto-N-biose phosphorylase, D-galactosyl-1,4-L-rhamnose phosphorylase |
|  | GH113 | 1 | 0 | β-mannanase |
|  | GH114 | 1 | 1 | endo-α-1,4-polygalactosaminidase |
|  | GH125 | 3 | 2 | exo-α-1,6-mannosidase |
|  | GH126 | 2 | 1 | amylase |
|  | GH127 | 0 | 3 | L-arabinofuranosidase, 3-C-carboxy-5-deoxy-L-xylose (aceric acid) hydrolase |
|  | GH129 | 1 | 0 | N-acetylgalactosaminidase |
|  | GH136 | 1 | 0 | lacto-N-biosidase |
|  | GH153 | 1 | 2 | β-1,6-D-glucosamine hydrolase |
|  | GH154 | 2 | 1 | β-glucuronidase |
|  | GH158 | 1 | 0 | endo-β-1,3-glucanase |
| GT | GT1 | 2 | 4 | 1-β-galactosyltransferase, salicylic acid β-glucosyltransferase, others |
|  | GT120 | 0 | 1 | O-antigen-polysaccharide α-1,3-L-rhamnosyltransferase |
|  | GT19 | 3 | 3 | lipid-A-disaccharide synthase |
|  | GT2 | 78 | 58 | cellulose synthase, chitin synthase, α-1,3-L-rhamnosyltransferase, others |
|  | GT20 | 2 | 2 | α,α-trehalose-phosphate synthase |
|  | GT25 | 1 | 0 | β-1,3-glucosyltransferase, β-1,2-glucosyltransferase, others |
|  | GT26 | 4 | 5 | β-N-acetyl mannosaminuronyltransferase, β-N-acetyl-mannosaminyltransferase, β-1,4-glucosyltransferase |
|  | GT27 | 0 | 2 | polypeptide α-N-acetylgalactosaminyltransferase |
|  | GT28 | 9 | 8 | 1,2-diacylglycerol 3-β-galactosyltransferase, others |
|  | GT30 | 2 | 1 | α-3-deoxy-D-manno-octulosonic-acid (KDO) transferase |
|  | GT32 | 2 | 0 | α-1,6-mannosyltransferase, α-1,4-N-acetylglucosaminyltransferase, α-1,4-N-acetylgalactosaminyltransferase |
|  | GT35 | 7 | 4 | glycogen or starch phosphorylase |
|  | GT39 | 0 | 1 | α-mannosyltransferase |
|  | GT4 | 45 | 34 | sucrose synthase, α-glucosyltransferase, α-mannosyltransferase, α-xylosyltransferase, others |
|  | GT41 | 0 | 1 | peptide β-N-acetylglucosaminyltransferase |
|  | GT5 | 3 | 3 | glycogen glucosyltransferase, α-1,3-glucan synthase, α-1,4-glucan synthase, others |
|  | GT51 | 20 | 18 | murein polymerase |
|  | GT56 | 2 | 1 | lipid II Fuc4NAc transferase |
|  | GT66 | 0 | 1 | β-oligosaccharyltransferase |
|  | GT73 | 2 | 0 | α-3-deoxy-D-manno-octulosonic-acid (KDO) transferase |
|  | GT76 | 0 | 2 | α-1,6-mannosyltransferase |
|  | GT8 | 13 | 4 | glycogenin glucosyltransferase, galactinol synthase, xylan α-glucuronyltransferase, others |
|  | GT81 | 1 | 0 | glucosyl-3-phosphoglycerate synthase, mannosyl-3-phosphoglycerate synthase |
|  | GT83 | 4 | 2 | 4-amino-4-deoxy-β-L-arabinosyltransferase, others |
|  | GT87 | 0 | 1 | α-1,2-mannosyltransferase |
|  | GT9 | 12 | 3 | lipopolysaccharide N-acetylglucosaminyltransferase, heptosyltransferase |
| PL | PL1 | 1 | 0 | pectate lyase, exo-pectate lyase, pectin lyase |
|  | PL12 | 0 | 1 | heparin-sulfate lyase |
|  | PL15 | 1 | 3 | oligo-alginate lyase |
|  | PL22 | 3 | 2 | oligogalacturonate lyase / oligogalacturonide lyase |
|  | PL31 | 1 | 0 | endo-β-1,4-glucuronan lyase |
|  | PL33 | 1 | 0 | hyaluronate lyase, gellan lyase, chondroitin sulfate lyase |
|  | PL8 | 1 | 2 | hyaluronate lyase, chondroitin AC lyase, xanthan lyase, chondroitin ABC lyase |
|  | PL9 | 5 | 2 | pectate lyase, exopolygalacturonate lyase, thiopeptidoglycan lyase |
|  |  |  |  |  |
| CBM | CBM13 | 1 | 0 | galactose residues-binding module |
|  | CBM14 | 0 | 1 | chitin-binding function |
|  | CBM16 | 2 | 0 | carbohydrate-binding module |
|  | CBM2 | 0 | 1 | cellulose-binding function |
|  | CBM26 | 1 | 0 | Starch-binding function |
|  | CBM28 | 0 | 1 | non-crystalline cellulose, cellooligosaccharides, and β-(1,3)(1,4)-glucans-binding function |
|  | CBM32 | 1 | 4 | galactose and lactose-binding module |
|  | CBM34 | 4 | 4 | granular starch-binding function |
|  | CBM35 | 1 | 0 | Cellvibrio xylan-degrading enzymes binds module |
|  | CBM37 | 0 | 1 | xylan, chitin, microcrystalline and phosphoric-acid swollen cellulose-binding module |
|  | CBM41 | 5 | 1 | α-glucans amylose, amylopectin, pullulan, and oligosaccharide fragments-binding module |
|  | CBM44 | 0 | 4 | cellulose and xyloglucan-binding module |
|  | CBM46 | 1 | 0 | Cellulose-binding function |
|  | CBM48 | 11 | 11 | glycogen-binding function |
|  | CBM5 | 2 | 3 | Chitin-binding module |
|  | CBM50 | 36 | 27 | Binding to chitopentaose |
|  | CBM51 | 1 | 0 | Binding to galactose |
|  | CBM56 | 0 | 1 | β-1,3-glucan binding function |
|  | CBM6 | 1 | 0 | The cellulose-binding function; bind β-1,3-glucan, β-1,3-1,4-glucan, and β-1,4-glucan. |
|  | CBM61 | 2 | 0 | β-1,4-galactan binding function |
|  | CBM66 | 2 | 0 | targets the terminal fructoside residue of fructans. |
|  | CBM67 | 3 | 1 | L-rhamnose binding activity |
|  | CBM68 | 0 | 1 | Binding to maltotriose and maltotetraose |
|  | CBM73 | 0 | 1 | Chitin-binding function |
|  | CBM85 | 1 | 0 | cellulose-binding function |
|  | CBM9 | 0 | 1 | cellulose-binding function |

**Table S7**. Significantly different metabolites in maize cellulose degradation products between different groups.

| Ionization mode | Metabolite | F.value | P.value | LOG10(p) | FDR | Tukey's HSD |
| --- | --- | --- | --- | --- | --- | --- |
| Positive ionization mode | Tetrahydrodipicolinate | 470.22 | 2.95E-14 | 13.531 | 1.05E-11 | BI-M-BI-D; CK-BI-D |
|  | Urocanic acid | 220.43 | 7.58E-12 | 11.121 | 1.35E-09 | BI-M-BI-D; CK-BI-D; CK-BI-M |
|  | 5-2-Hydroxyethyl-4-methylthiazole | 115.68 | 7.66E-10 | 9.1161 | 9.11E-08 | BI-M-BI-D; CK-BI-D |
|  | Putrescine | 110.19 | 1.08E-09 | 8.9677 | 9.61E-08 | BI-M-BI-D; CK-BI-D |
|  | Deoxyguanosine | 99.399 | 2.22E-09 | 8.6543 | 1.58E-07 | BI-M-BI-D; CK-BI-D |
|  | 10E,12Z-Octadecadienoic acid | 75.782 | 1.44E-08 | 7.8412 | 8.58E-07 | BI-M-BI-D; CK-BI-D |
|  | Tryptophanol | 72.014 | 2.04E-08 | 7.6904 | 1.04E-06 | BI-M-BI-D; CK-BI-D |
|  | Histamine | 63.298 | 4.87E-08 | 7.3122 | 2.17E-06 | BI-M-BI-D; CK-BI-D; CK-BI-M |
|  | N-Acetylhistamine | 53.636 | 1.46E-07 | 6.8343 | 5.81E-06 | BI-M-BI-D; CK-BI-M |
|  | cis-9,10-Epoxystearic acid | 51.481 | 1.92E-07 | 6.7174 | 6.70E-06 | BI-M-BI-D; CK-BI-D |
|  | Cytidine | 50.903 | 2.06E-07 | 6.6853 | 6.70E-06 | BI-M-BI-D; CK-BI-D |
|  | L-3-Cyanoalanine | 43.179 | 5.98E-07 | 6.2232 | 1.78E-05 | CK-BI-D; CK-BI-M |
|  | S-N-Methylcoclaurine | 39.925 | 9.84E-07 | 6.0071 | 2.70E-05 | BI-M-BI-D; CK-BI-D |
|  | 2-Phenylethanol | 39.371 | 1.07E-06 | 5.9688 | 2.74E-05 | BI-M-BI-D; CK-BI-D |
|  | Prostaglandin D2 | 38.64 | 1.21E-06 | 5.9177 | 2.88E-05 | BI-M-BI-D; CK-BI-D |
|  | Isocorypalmine | 33.858 | 2.75E-06 | 5.5612 | 6.13E-05 | BI-M-BI-D; CK-BI-D; CK-BI-M |
|  | N6-Acetyl-L-lysine | 33.363 | 3.01E-06 | 5.522 | 6.31E-05 | BI-M-BI-D; CK-BI-D |
|  | 3-Methylindole | 29.349 | 6.53E-06 | 5.1853 | 0.000129 | BI-M-BI-D; CK-BI-D; CK-BI-M |
|  | Normetanephrine | 29.045 | 6.95E-06 | 5.1583 | 0.000131 | BI-M-BI-D; CK-BI-D |
|  | Cyclopropanecarboxylate | 26.858 | 1.10E-05 | 4.9573 | 0.000197 | BI-M-BI-D; CK-BI-D; CK-BI-M |
|  | Eicosadienoic acid | 26.518 | 1.19E-05 | 4.9248 | 0.000202 | BI-M-BI-D; CK-BI-D; CK-BI-M |
|  | N-Acetylputrescine | 25.407 | 1.53E-05 | 4.8167 | 0.000238 | CK-BI-D; CK-BI-M |
|  | Alpha-Linolenic acid | 25.247 | 1.58E-05 | 4.8009 | 0.000238 | BI-M-BI-D; CK-BI-D; CK-BI-M |
|  | Glycerophosphocholine | 25.046 | 1.66E-05 | 4.7807 | 0.000238 | CK-BI-D; CK-BI-M |
|  | Indole | 25.016 | 1.67E-05 | 4.7778 | 0.000238 | BI-M-BI-D; CK-BI-M |
|  | Chlorogenic acid | 24.728 | 1.78E-05 | 4.7488 | 0.000245 | BI-M-BI-D; CK-BI-D; CK-BI-M |
|  | 3-Methyl-2-butenal | 24.36 | 1.94E-05 | 4.7114 | 0.000257 | BI-M-BI-D; CK-BI-D |
|  | Oxalureate | 23.397 | 2.45E-05 | 4.6114 | 0.000293 | BI-M-BI-D; CK-BI-M |
|  | Indoleacetaldehyde | 23.382 | 2.46E-05 | 4.6099 | 0.000293 | CK-BI-D; CK-BI-M |
|  | Ornithine | 23.377 | 2.46E-05 | 4.6093 | 0.000293 | CK-BI-D; CK-BI-M |
|  | Penicillin N | 22.884 | 2.77E-05 | 4.5569 | 0.000319 | BI-M-BI-D; CK-BI-D; CK-BI-M |
|  | Tryptamine | 22.287 | 3.22E-05 | 4.4922 | 0.000359 | BI-M-BI-D; CK-BI-D; CK-BI-M |
|  | 5-Methylcytosine | 21.277 | 4.17E-05 | 4.3799 | 0.000451 | BI-M-BI-D; CK-BI-D; CK-BI-M |
|  | Lumichrome | 20.624 | 4.95E-05 | 4.3051 | 0.000517 | BI-M-BI-D; CK-BI-M |
|  | 2-Hydroxybutyric acid | 20.47 | 5.16E-05 | 4.2873 | 0.000517 | BI-M-BI-D; CK-BI-D; CK-BI-M |
|  | Pyrimidodiazepine | 20.431 | 5.22E-05 | 4.2827 | 0.000517 | BI-M-BI-D; CK-BI-M |
|  | Scolymoside | 20.173 | 5.59E-05 | 4.2525 | 0.000539 | BI-M-BI-D; CK-BI-M |
|  | Cellobiose | 19.358 | 7.00E-05 | 4.1551 | 0.000657 | CK-BI-D; CK-BI-M |
|  | Mesaconate | 19.263 | 7.19E-05 | 4.1436 | 0.000658 | BI-M-BI-D; CK-BI-D |
|  | Piperidine | 18.427 | 9.12E-05 | 4.0402 | 0.000814 | BI-M-BI-D; CK-BI-D |
|  | L-Threonine | 17.93 | 0.000105 | 3.9771 | 0.000918 | CK-BI-D; CK-BI-M |
|  | 3D-3,5/4-Trihydroxycyclohexane-1,2-dione | 17.821 | 0.000109 | 3.9632 | 0.000925 | BI-M-BI-D; CK-BI-D; CK-BI-M |
|  | Serotonin | 16.843 | 0.000146 | 3.8348 | 0.001214 | BI-M-BI-D; CK-BI-M |
|  | Guanosine 2,3-cyclic phosphate | 16.022 | 0.000189 | 3.723 | 0.001535 | CK-BI-D; CK-BI-M |
|  | N-Formyl-L-glutamic acid | 15.884 | 0.000198 | 3.7039 | 0.001569 | BI-M-BI-D; CK-BI-M |
|  | Guanine | 15.572 | 0.000219 | 3.6601 | 0.001697 | BI-M-BI-D; CK-BI-D |
|  | L-erythro-4-Hydroxyglutamate | 14.84 | 0.000279 | 3.5551 | 0.002116 | BI-M-BI-D; CK-BI-D; CK-BI-M |
|  | Cyclic AMP | 14.698 | 0.000292 | 3.5345 | 0.002173 | CK-BI-D; CK-BI-M |
|  | Nicotinamide riboside | 14.486 | 0.000314 | 3.5031 | 0.002288 | CK-BI-D; CK-BI-M |
|  | Geranyl diphosphate | 13.925 | 0.000381 | 3.419 | 0.002721 | CK-BI-D; CK-BI-M |
|  | Xanthoxic acid | 13.487 | 0.000445 | 3.3517 | 0.003002 | BI-M-BI-D; CK-BI-M |
|  | L-Glutamic acid | 13.455 | 0.00045 | 3.3467 | 0.003002 | CK-BI-D; CK-BI-M |
|  | D-Lombricine | 13.426 | 0.000455 | 3.3422 | 0.003002 | BI-M-BI-D; CK-BI-D |
|  | Hypoxanthine | 13.419 | 0.000456 | 3.341 | 0.003002 | CK-BI-D; CK-BI-M |
|  | Anatabine | 13.379 | 0.000462 | 3.3349 | 0.003002 | BI-M-BI-D; CK-BI-M |
|  | Perillyl alcohol | 13.301 | 0.000476 | 3.3226 | 0.003033 | CK-BI-D |
|  | 4-Guanidinobutanoic acid | 13.197 | 0.000494 | 3.3063 | 0.003094 | BI-M-BI-D; CK-BI-M |
|  | Indoleacetic acid | 13.023 | 0.000526 | 3.2789 | 0.003238 | BI-M-BI-D; CK-BI-D |
|  | Cholesterol | 12.74 | 0.000584 | 3.2336 | 0.003533 | BI-M-BI-D; CK-BI-M |
|  | 2-Methylbenzoic acid | 12.687 | 0.000596 | 3.2251 | 0.003543 | BI-M-BI-D; CK-BI-M |
|  | Guanosine | 12.371 | 0.00067 | 3.1738 | 0.003923 | BI-M-BI-D; CK-BI-M |
|  | beta-Alanyl-L-lysine | 12.089 | 0.000746 | 3.1271 | 0.004297 | CK-BI-M |
|  | Trehalose | 11.978 | 0.000779 | 3.1086 | 0.004413 | BI-M-BI-D; CK-BI-D |
|  | Estragole | 11.892 | 0.000805 | 3.0942 | 0.004457 | BI-M-BI-D; CK-BI-D |
|  | 2-Deoxyadenosine | 11.871 | 0.000812 | 3.0907 | 0.004457 | BI-M-BI-D; CK-BI-M |
|  | beta-Alanine | 11.683 | 0.000873 | 3.059 | 0.004689 | BI-M-BI-D; CK-BI-M |
|  | Phenylacetaldehyde | 11.663 | 0.00088 | 3.0555 | 0.004689 | BI-M-BI-D; CK-BI-D |
|  | N-Acetyl-O-demethylpuromycin-5-phosphate | 11.451 | 0.000957 | 3.0193 | 0.005022 | BI-M-BI-D; CK-BI-D |
|  | Coniferyl aldehyde | 11.248 | 0.001037 | 2.9843 | 0.005365 | BI-M-BI-D; CK-BI-M |
|  | DL-Dopa | 11.189 | 0.001062 | 2.9739 | 0.005415 | BI-M-BI-D; CK-BI-M |
|  | 3-Methoxytyramine | 10.826 | 0.00123 | 2.91 | 0.006187 | BI-M-BI-D; CK-BI-M |
|  | Deoxycholic acid | 10.716 | 0.001287 | 2.8904 | 0.006251 | BI-M-BI-D; CK-BI-M |
|  | Saccharopine | 10.709 | 0.001291 | 2.8891 | 0.006251 | BI-M-BI-D |
|  | gamma-L-Glutamyl-L-2-aminobutyrate | 10.698 | 0.001297 | 2.8872 | 0.006251 | CK-BI-D; CK-BI-M |
|  | Ubiquinone-1 | 10.663 | 0.001316 | 2.8809 | 0.006251 | CK-BI-D; CK-BI-M |
|  | Adenine | 10.635 | 0.001331 | 2.8759 | 0.006251 | BI-M-BI-D; CK-BI-M |
|  | Cadaverine | 10.577 | 0.001363 | 2.8655 | 0.00632 | CK-BI-D; CK-BI-M |
|  | Cytosine | 10.518 | 0.001397 | 2.8548 | 0.006378 | BI-M-BI-D; CK-BI-M |
|  | L-Kynurenine | 10.493 | 0.001411 | 2.8504 | 0.006378 | CK-BI-D; CK-BI-M |
|  | Biotin | 10.362 | 0.001491 | 2.8265 | 0.006653 | BI-M-BI-D; CK-BI-M |
|  | 5-KETE | 10.248 | 0.001564 | 2.8056 | 0.006895 | BI-M-BI-D; CK-BI-D |
|  | 5-Hydroxyindolepyruvate | 10.209 | 0.00159 | 2.7986 | 0.006923 | BI-M-BI-D; CK-BI-D |
|  | D-Fructose | 10.146 | 0.001633 | 2.7869 | 0.007026 | CK-BI-M |
|  | 5-Hydroxyindoleacetic acid | 10.118 | 0.001653 | 2.7817 | 0.007026 | BI-M-BI-D; CK-BI-M |
|  | Citraconic acid | 10.039 | 0.00171 | 2.767 | 0.007054 | BI-M-BI-D |
|  | Indolepyruvate | 10.021 | 0.001723 | 2.7637 | 0.007054 | CK-BI-D; CK-BI-M |
|  | 3-Methoxyanthranilate | 10.018 | 0.001725 | 2.7632 | 0.007054 | CK-BI-D; CK-BI-M |
|  | Cytisine | 9.9996 | 0.001739 | 2.7597 | 0.007054 | BI-M-BI-D; CK-BI-D |
|  | D-Maltose | 9.9499 | 0.001776 | 2.7505 | 0.007089 | CK-BI-D; CK-BI-M |
|  | Phthalic acid | 9.9357 | 0.001787 | 2.7478 | 0.007089 | BI-M-BI-D; CK-BI-M |
|  | Leucodopachrome | 9.8581 | 0.001848 | 2.7333 | 0.00725 | CK-BI-D; CK-BI-M |
|  | 15-Keto-prostaglandin F2a | 9.6498 | 0.002023 | 2.694 | 0.007851 | CK-BI-D; CK-BI-M |
|  | Uracil | 9.5947 | 0.002073 | 2.6835 | 0.007956 | CK-BI-D; CK-BI-M |
|  | L-Isoleucine | 9.4291 | 0.00223 | 2.6518 | 0.008467 | BI-M-BI-D |
|  | N-Acetyl-L-glutamate 5-semialdehyde | 9.2315 | 0.002435 | 2.6136 | 0.009102 | BI-M-BI-D; CK-BI-D |
|  | R-3-Hydroxybutyric acid | 9.2196 | 0.002448 | 2.6112 | 0.009102 | CK-BI-D; CK-BI-M |
|  | 2S,5S-trans-Carboxymethylproline | 9.0888 | 0.002596 | 2.5856 | 0.009555 | CK-BI-D; CK-BI-M |
|  | S-Ribosyl-L-homocysteine | 8.912 | 0.002814 | 2.5508 | 0.010249 | CK-BI-D |
|  | UMP | 8.8391 | 0.002909 | 2.5363 | 0.01049 | CK-BI-D; CK-BI-M |
|  | Niacinamide | 8.748 | 0.003034 | 2.518 | 0.010823 | BI-M-BI-D; CK-BI-D |
|  | N-Acetylserotonin | 8.7278 | 0.003062 | 2.514 | 0.010823 | BI-M-BI-D; CK-BI-M |
|  | 7-Aminomethyl-7-carbaguanine | 8.7014 | 0.0031 | 2.5087 | 0.010849 | BI-M-BI-D; CK-BI-M |
|  | N-Butyryl-L-homoserine lactone | 8.6031 | 0.003244 | 2.4889 | 0.011245 | BI-M-BI-D; CK-BI-D |
|  | Benzamide | 8.5775 | 0.003283 | 2.4837 | 0.011271 | BI-M-BI-D; CK-BI-M |
|  | Geranic acid | 8.5217 | 0.00337 | 2.4724 | 0.011458 | BI-M-BI-D; CK-BI-M |
|  | gamma-Glutamylalanine | 8.4651 | 0.003461 | 2.4608 | 0.011656 | CK-BI-D; CK-BI-M |
|  | Phosphoserine | 8.4043 | 0.003561 | 2.4484 | 0.011832 | CK-BI-D; CK-BI-M |
|  | Phenylacetic acid | 8.3935 | 0.003579 | 2.4462 | 0.011832 | BI-M-BI-D; CK-BI-D |
|  | trans-Ferulic acid | 8.2801 | 0.003777 | 2.4229 | 0.01237 | CK-BI-D; CK-BI-M |
|  | Quercetin | 8.2389 | 0.003852 | 2.4144 | 0.0125 | BI-M-BI-D; CK-BI-M |
|  | Tetrahydrobiopterin | 8.1268 | 0.004064 | 2.3911 | 0.01307 | BI-M-BI-D |
|  | Riboflavin | 8.0267 | 0.004265 | 2.3701 | 0.013593 | CK-BI-D |
|  | L-Rhamnofuranose | 7.931 | 0.004467 | 2.35 | 0.014112 | CK-BI-D |
|  | 1,2,3-Trihydroxybenzene | 7.836 | 0.004679 | 2.3299 | 0.014419 | BI-M-BI-D; CK-BI-D |
|  | 4-Hydroxybutanoic acid | 7.8339 | 0.004684 | 2.3294 | 0.014419 | BI-M-BI-D |
|  | Xanthine | 7.8331 | 0.004685 | 2.3293 | 0.014419 | CK-BI-M |
|  | N-Acetylornithine | 7.712 | 0.004972 | 2.3034 | 0.015172 | CK-BI-D; CK-BI-M |
|  | Vanillin | 7.5157 | 0.005481 | 2.2611 | 0.016546 | BI-M-BI-D |
|  | Cyclic GMP | 7.5033 | 0.005515 | 2.2584 | 0.016546 | BI-M-BI-D; CK-BI-M |
|  | Pelargonin | 7.4382 | 0.005698 | 2.2443 | 0.016952 | BI-M-BI-D; CK-BI-M |
|  | Dopamine | 7.3541 | 0.005944 | 2.2259 | 0.017453 | BI-M-BI-D |
|  | O-Acetylserine | 7.3474 | 0.005965 | 2.2244 | 0.017453 | CK-BI-D |
|  | D-Octopine | 7.3184 | 0.006053 | 2.218 | 0.017568 | BI-M-BI-D; CK-BI-M |
|  | Imidazoleacetic acid | 7.2078 | 0.006403 | 2.1937 | 0.018433 | CK-BI-D |
|  | Pelletierine | 7.1894 | 0.006463 | 2.1896 | 0.018458 | BI-M-BI-D |
|  | Sinapoyl aldehyde | 7.0712 | 0.006867 | 2.1632 | 0.019456 | BI-M-BI-D; CK-BI-M |
|  | S-Adenosylmethionine | 6.8619 | 0.007654 | 2.1161 | 0.021515 | CK-BI-D |
|  | 1-Amino-1-deoxy-scyllo-inositol | 6.8284 | 0.007789 | 2.1085 | 0.021724 | CK-BI-D; CK-BI-M |
|  | 1-Pyrroline-2-carboxylic acid | 6.7833 | 0.007975 | 2.0983 | 0.022071 | BI-M-BI-D |
|  | Vanillylmandelic acid | 6.5879 | 0.008843 | 2.0534 | 0.024285 | BI-M-BI-D; CK-BI-M |
|  | 4-Hydroxy-2-oxoglutaric acid | 6.4894 | 0.009321 | 2.0305 | 0.025401 | BI-M-BI-D; CK-BI-M |
|  | 8-Isoprostane | 6.4182 | 0.009685 | 2.0139 | 0.026192 | BI-M-BI-D; CK-BI-D |
|  | Oxoadipic acid | 6.3775 | 0.0099 | 2.0044 | 0.026573 | BI-M-BI-D; CK-BI-M |
|  | Benzoate | 6.1583 | 0.011155 | 1.9525 | 0.02972 | CK-BI-D |
|  | N2-Succinyl-L-arginine | 6.1364 | 0.011291 | 1.9473 | 0.029858 | CK-BI-M |
|  | Phytosphingosine | 6.0842 | 0.01162 | 1.9348 | 0.030502 | CK-BI-D |
|  | 4-Hydroxystyrene | 6.018 | 0.012054 | 1.9189 | 0.03141 | CK-BI-M |
|  | Anserine | 5.9792 | 0.012316 | 1.9095 | 0.031861 | CK-BI-D |
|  | Phenol | 5.9564 | 0.012474 | 1.904 | 0.031954 | CK-BI-M |
|  | trans-trans-Muconic acid | 5.9482 | 0.012531 | 1.902 | 0.031954 | BI-M-BI-D; CK-BI-D |
|  | 1H-Indole-3-acetamide | 5.9073 | 0.012821 | 1.8921 | 0.032461 | BI-M-BI-D |
|  | Ophthalmate | 5.7782 | 0.013786 | 1.8606 | 0.034624 | CK-BI-D |
|  | 3,4-Dihydroxybenzaldehyde | 5.7675 | 0.013869 | 1.858 | 0.034624 | BI-M-BI-D; CK-BI-M |
|  | 6-Hydroxymelatonin | 5.6697 | 0.01466 | 1.8339 | 0.036345 | BI-M-BI-D; CK-BI-M |
|  | Epinephrine | 5.6074 | 0.015191 | 1.8184 | 0.037156 | BI-M-BI-D |
|  | trans-Aconitic acid | 5.6069 | 0.015195 | 1.8183 | 0.037156 | CK-BI-M |
|  | Pipecolic acid | 5.5406 | 0.015785 | 1.8018 | 0.038305 | CK-BI-D; CK-BI-M |
|  | L-Arabinonate | 5.5301 | 0.01588 | 1.7991 | 0.038305 | CK-BI-M |
|  | p-Anisic acid | 5.4897 | 0.016254 | 1.789 | 0.038944 | BI-M-BI-D |
|  | 2-Amino-2-deoxy-D-gluconate | 5.4603 | 0.016533 | 1.7817 | 0.039348 | CK-BI-D |
|  | 5-Hydroxylysine | 5.3989 | 0.017132 | 1.7662 | 0.040504 | BI-M-BI-D; CK-BI-M |
|  | 8-Amino-7-oxononanoate | 5.3662 | 0.017462 | 1.7579 | 0.041012 | BI-M-BI-D; CK-BI-D |
|  | trans-Isopiperitenol | 5.2429 | 0.018769 | 1.7266 | 0.043546 | CK-BI-D |
|  | 4-Hydroxyphenylacetaldehyde | 5.2415 | 0.018785 | 1.7262 | 0.043546 | BI-M-BI-D |
|  | L-Lysine | 5.2103 | 0.019134 | 1.7182 | 0.044 | CK-BI-D; CK-BI-M |
|  | Coniferyl alcohol | 5.202 | 0.019227 | 1.7161 | 0.044 | CK-BI-D |
|  | Beta-D-Glucose | 5.1317 | 0.020044 | 1.698 | 0.045445 | BI-M-BI-D |
|  | Uric acid | 5.126 | 0.020113 | 1.6965 | 0.045445 | CK-BI-M |
|  | 3S,5S-Carbapenam-3-carboxylic acid | 5.0494 | 0.021052 | 1.6767 | 0.047267 | BI-M-BI-D |
| Negative ionization mode | Stearic acid | 180.59 | 3.20E-11 | 10.495 | 4.32E-09 | BI-M-BI-D; CK-BI-M |
|  | 3,4-Dihydroxyhydrocinnamic acid | 134.45 | 2.64E-10 | 9.5781 | 1.78E-08 | BI-M-BI-D; CK-BI-D |
|  | L-Tryptophan | 43.127 | 6.03E-07 | 6.2199 | 2.71E-05 | BI-M-BI-D; CK-BI-D; CK-BI-M |
|  | L-Histidine | 40.595 | 8.86E-07 | 6.0528 | 2.99E-05 | BI-M-BI-D; CK-BI-D; CK-BI-M |
|  | Malvidin 3-glucoside | 32.072 | 3.82E-06 | 5.4175 | 0.000103 | BI-M-BI-D; CK-BI-D; CK-BI-M |
|  | Indolepyruvate | 29.855 | 5.89E-06 | 5.2296 | 0.000133 | BI-M-BI-D; CK-BI-D; CK-BI-M |
|  | Isowertin 2-rhamnoside | 28.681 | 7.49E-06 | 5.1257 | 0.000144 | BI-M-BI-D; CK-BI-D |
|  | Naringenin | 20.929 | 4.57E-05 | 4.3403 | 0.000753 | BI-M-BI-D; CK-BI-D; CK-BI-M |
|  | Isovitexin | 20.441 | 5.20E-05 | 4.2839 | 0.000753 | BI-M-BI-D; CK-BI-D |
|  | N6-Delta2-Isopentenyl-adenine | 19.866 | 6.08E-05 | 4.2161 | 0.000753 | BI-M-BI-D; CK-BI-D |
|  | Gluconic acid | 19.522 | 6.69E-05 | 4.1749 | 0.000753 | BI-M-BI-D; CK-BI-M |
|  | Hesperetin | 19.515 | 6.70E-05 | 4.1741 | 0.000753 | CK-BI-D; CK-BI-M |
|  | S-Adenosylmethioninamine | 18.164 | 9.84E-05 | 4.007 | 0.001022 | BI-M-BI-D; CK-BI-D |
|  | Luteolin | 17.622 | 0.000115 | 3.9375 | 0.001077 | BI-M-BI-D; CK-BI-M |
|  | Chorismate | 17.503 | 0.00012 | 3.922 | 0.001077 | BI-M-BI-D; CK-BI-M |
|  | 4-Hydroxycinnamyl alcohol 4-D-glucoside | 17.283 | 0.000128 | 3.8932 | 0.001079 | BI-M-BI-D; CK-BI-M |
|  | 2-Pyrocatechuic acid | 17.011 | 0.000139 | 3.8572 | 0.001103 | BI-M-BI-D; CK-BI-M |
|  | Protoemetine | 16.135 | 0.000183 | 3.7387 | 0.001369 | BI-M-BI-D; CK-BI-D |
|  | cis-4-Hydroxy-D-proline | 14.342 | 0.00033 | 3.4818 | 0.002343 | BI-M-BI-D; CK-BI-M |
|  | Metanephrine | 13.472 | 0.000447 | 3.3493 | 0.00302 | BI-M-BI-D; CK-BI-M |
|  | D-Glucurono-6,3-lactone | 13.322 | 0.000472 | 3.326 | 0.003035 | BI-M-BI-D; CK-BI-D |
|  | Sedoheptulose | 13.088 | 0.000514 | 3.2891 | 0.003154 | BI-M-BI-D; CK-BI-M |
|  | Sorbitol | 12.766 | 0.000578 | 3.2378 | 0.003394 | BI-M-BI-D; CK-BI-D |
|  | N-Formyl-L-methionine | 12.573 | 0.000621 | 3.2067 | 0.003495 | BI-M-BI-D; CK-BI-D |
|  | Oxoglutaric acid | 12.001 | 0.000772 | 3.1124 | 0.004168 | BI-M-BI-D; CK-BI-D |
|  | 5-Methylthioadenosine | 11.009 | 0.001142 | 2.9424 | 0.005929 | CK-BI-D; CK-BI-M |
|  | Isomaltose | 10.702 | 0.001294 | 2.888 | 0.006319 | BI-M-BI-D; CK-BI-D |
|  | 3-O-Methylquercetin | 10.672 | 0.001311 | 2.8825 | 0.006319 | BI-M-BI-D; CK-BI-M |
|  | 2-Aminobenzoic acid | 10.512 | 0.0014 | 2.8538 | 0.006499 | CK-BI-D |
|  | Norepinephrine | 10.416 | 0.001458 | 2.8364 | 0.006499 | BI-M-BI-D; CK-BI-M |
|  | 3-Methylxanthine | 10.36 | 0.001492 | 2.8261 | 0.006499 | BI-M-BI-D; CK-BI-M |
|  | Mannitol | 9.7273 | 0.001956 | 2.7087 | 0.008251 | CK-BI-D; CK-BI-M |
|  | 4-Nitrophenol | 9.1641 | 0.00251 | 2.6004 | 0.010266 | CK-BI-D; CK-BI-M |
|  | 3-3,4-Dihydroxy-5-methoxy-2-propenoic acid | 8.5689 | 0.003297 | 2.4819 | 0.013089 | CK-BI-M |
|  | 4-Hydroxyphenylpyruvic acid | 8.3457 | 0.003661 | 2.4364 | 0.014008 | BI-M-BI-D; CK-BI-M |
|  | Deoxyguanosine | 8.3033 | 0.003736 | 2.4276 | 0.014008 | BI-M-BI-D; CK-BI-D |
|  | 5-Hydroxy-3-indoleaceacetate | 7.9353 | 0.004458 | 2.3509 | 0.016264 | BI-M-BI-D; CK-BI-D |
|  | Citric acid | 7.5128 | 0.005489 | 2.2605 | 0.0195 | BI-M-BI-D; CK-BI-M |
|  | Divinyl chlorophyllide a | 7.1324 | 0.006654 | 2.1769 | 0.023033 | CK-BI-M |
|  | Ribitol | 6.8765 | 0.007596 | 2.1194 | 0.025635 | BI-M-BI-D |
|  | Apiin | 6.2962 | 0.010346 | 1.9852 | 0.034065 | BI-M-BI-D |
|  | Ascorbate | 6.1534 | 0.011186 | 1.9513 | 0.035954 | BI-M-BI-D; CK-BI-M |
|  | Caffeine | 6.0096 | 0.01211 | 1.9169 | 0.038019 | BI-M-BI-D |
|  | Syringic acid | 5.8248 | 0.013428 | 1.872 | 0.040384 | BI-M-BI-D |
|  | Protochlorophyllide | 5.7867 | 0.01372 | 1.8627 | 0.040384 | BI-M-BI-D |
|  | Pantetheine 4-phosphate | 5.7814 | 0.013761 | 1.8614 | 0.040384 | BI-M-BI-D; CK-BI-M |
|  | Aminoadipic acid | 5.5195 | 0.015977 | 1.7965 | 0.045892 | CK-BI-M |
|  | 12-Ethyl-8-isobutyl-3-vinylbacteriochlorophyllide d | 5.4494 | 0.016637 | 1.7789 | 0.046337 | BI-M-BI-D |
|  | Sinapic acid | 5.4307 | 0.016819 | 1.7742 | 0.046337 | CK-BI-M |
|  | Gentisic acid | 5.28 | 0.018364 | 1.736 | 0.049271 | BI-M-BI-D |
|  | Itaconic acid | 5.2571 | 0.018614 | 1.7302 | 0.049271 | BI-M-BI-D; CK-BI-D |
